# Prenatal Diagnosis and Perinatal Management Considerations in Congenital Abdominal Wall Defects: A Case Series and Narrative Review

**DOI:** 10.3390/jpm16090460

**Published:** 2026-08-31

**Authors:** Nikola Popovski, Nikoleta Stoyanova, Rebecca Caiulo

**Affiliations:** 1 Department of Obstetrics and Gynecology, Medical University Pleven, 5800 Pleven, Bulgaria; n_popovsky@abv.bg (N.P.); niikoletastoyanova@gmail.com (N.S.); 2 Clinics of Obstetrics and Gynecology, UMHAT “Dr. Georgi Stranski”, 5800 Pleven, Bulgaria

**Keywords:** gastroschisis, omphalocele, body stalk anomaly, prenatal diagnosis, fetal ultrasound, perinatal management, delivery planning

## Abstract

**Background**: Congenital abdominal wall defects (CAWDs), primarily gastroschisis and omphalocele, result from disturbances in early embryonic folding and midgut development and are routinely detected during prenatal ultrasound screening. Despite advances in prenatal imaging, considerable heterogeneity in clinical presentation, severity, and outcomes necessitates structured risk stratification to optimize prenatal and perinatal management. **Objectives**: We aimed to present a series of prenatally diagnosed congenital abdominal wall defects and integrate current evidence into a clinically applicable, risk-adapted framework for prenatal assessment and perinatal management. **Materials and Methods**: Three pathological cases of CAWDs diagnosed between 2025 and 2026 were retrospectively analyzed. Two additional first-trimester ultrasound examinations demonstrating physiological midgut herniation were included as illustrative examples of an important differential diagnosis during early pregnancy. Prenatal assessment included systematic evaluation of bowel dilatation, bowel wall thickness, liver herniation, and associated structural anomalies. Established risk stratification systems—including the distinction between simple and complex gastroschisis and the classification of omphalocele according to defect size and associated anomalies—were applied. A narrative review of the literature was performed to contextualize the clinical findings and support the development of a practical ultrasound-based diagnostic and management algorithm. **Results**: The pathological cases illustrated the broad clinical spectrum of CAWDs, ranging from isolated omphalocele to lethal body stalk anomaly, while the illustrative examples emphasized the importance of distinguishing physiological midgut herniation from pathological abdominal wall defects during the first trimester. Prenatal risk stratification based on ultrasound findings may inform surveillance strategies, delivery planning, and parental counseling. In particular, associated anomalies and liver herniation in omphalocele, as well as progressive bowel abnormalities in gastroschisis, were identified as key determinants of prognosis and clinical management. Based on the literature review and the illustrative institutional cases, an educational ultrasound-based diagnostic and management framework was proposed to summarize the current evidence and support a structured diagnostic approach. **Conclusions**: Congenital abdominal wall defects should be considered a spectrum of disorders with varying embryological origins, clinical manifestations, and prognostic implications. A standardized prenatal assessment combined with risk-adapted management can improve prognostic accuracy, optimize perinatal planning, and support informed parental counseling. Implementation of structured diagnostic frameworks may enhance clinical decision making and improve outcomes in affected pregnancies.

## 1. Introduction

Congenital abdominal wall defects (CAWDs) are a spectrum of developmental anomalies resulting from partial or complete failure of closure of the anterior abdominal wall, leading to herniation of abdominal—or, in severe cases, thoracic—organs outside the fetal body cavity. These defects arise during early embryonic development and exhibit considerable heterogeneity in severity, associated structural or chromosomal abnormalities, and postnatal outcomes, thus making them a clinically significant group of conditions within prenatal diagnosis and perinatal care.

CAWDs hold particular importance in contemporary obstetric practice because they are frequently detected during routine first- or second-trimester ultrasonography. Early identification has major implications for parental counseling, genetic evaluation, and perinatal planning. Gastroschisis and omphalocele account for the majority of entities within this group and differ substantially in their embryologic origins, associated anomalies, and expected clinical course [1].

In addition to these well-established entities, several conditions may enter the differential diagnosis of abdominal wall-related findings on prenatal imaging, requiring careful anatomical and embryological distinction. According to Ciopraga et al. [2], fetal inguinal hernia should be distinguished from CAWDs. While CAWDs, including gastroschisis and omphalocele, result from failure of anterior abdominal wall closure during embryogenesis, congenital inguinal hernia arises from persistence of the processus vaginalis and represents a defect of inguinal canal closure rather than primary abdominal wall formation. Unlike CAWDs, fetal inguinal hernia does not involve a true defect of the anterior abdominal wall musculature and typically presents as herniation of abdominal contents through the inguinal canal, most commonly during the third trimester. Accurate prenatal differentiation is essential for appropriate parental counseling, multidisciplinary perinatal planning, and postnatal surgical management.

Gastroschisis typically presents as an isolated right-sided paraumbilical full thickness defect, leaving the bowel loops free-floating within the amniotic cavity, and carries risk of progressive intrauterine bowel injury, fetal growth restriction, and postnatal complications [3]. In contrast, omphalocele involves herniated viscera enclosed within a membranous sac and is frequently associated with chromosomal abnormalities, cardiac defects, and syndromic diagnoses, necessitating comprehensive prenatal evaluation. More severe and less common anomalies, such as body stalk anomaly (BSA) and limb–body wall complex (LBWC), reflect early disruptions in embryonic folding and are associated with uniformly poor prognosis, underscoring the importance of accurate prenatal differentiation.

As detection rates rise and prenatal imaging continues to advance, understanding the epidemiology, diagnostic pathways, and clinical implications of CAWDs has become increasingly important. A clear framework for distinguishing among these defects is essential for optimizing prenatal counseling, anticipating perinatal needs, and coordinating multidisciplinary care.

Against this background, we present three pathological cases of prenatally diagnosed CAWDs, complemented by two illustrative examples of physiological midgut herniation, to highlight practical considerations in prenatal assessment and perinatal management.

## 2. Methodology

This manuscript combines a retrospective case series with a structured narrative review of CAWDs, including gastroschisis, omphalocele, and BSA. The aim is to summarize current evidence on prenatal diagnosis, imaging features, risk stratification, and perinatal management strategies, integrating these findings with clinical cases.

To illustrate the spectrum of CAWDs and their clinical implications, we present three pathological cases managed at our University Multiprofile Hospital between 2025 and 2026. In addition, two first-trimester ultrasound examinations demonstrating physiological midgut herniation are included as illustrative examples of a normal embryological finding that represents an important differential diagnosis of omphalocele during early pregnancy. Representative imaging findings are presented to emphasize key diagnostic features relevant to prenatal assessment.

In addition, we conducted a narrative literature review based on a structured search of the PubMed and Google Scholar databases. The search included combinations of keywords such as “congenital abdominal wall defects”, “omphalocele”, “gastroschisis”, and “body stalk anomaly.” Relevant articles published in English were selected based on their clinical relevance and contribution to the understanding of prenatal diagnosis, associated anomalies, and perinatal outcomes.

This narrative review of CAWDs summarizes and critically appraises the current knowledge on CAWDs, focusing on risk factors, diagnostic criteria, and management strategies. Although not conducted as a systematic review, the search and selection process followed a structured and transparent approach to ensure comprehensive coverage of the available literature.

### 2.1. Literature Search Strategy

In light of the heterogeneity of CAWDs, variability in prenatal diagnostic criteria, and lack of standardized management protocols, a structured literature search was conducted in PubMed and Google Scholar for studies published between 1 January 1981 and 1 May 2026. The primary search string used in PubMed was (“gastroschisis” OR “omphalocele” OR “body stalk anomaly” OR “congenital abdominal wall defect” OR “fetal abdominal wall defect”) AND (prenatal diagnosis OR ultrasound OR MRI OR Doppler OR management OR outcome OR delivery). A similar strategy was applied in Google Scholar to identify additional relevant publications not indexed in PubMed. Given the nature of Google Scholar indexing, this search was used primarily for citation chaining and supplementary retrieval of relevant studies. Reference lists of included articles and relevant reviews were hand-screened to identify additional eligible studies.

### 2.2. Eligibility Criteria

Studies were included if they met the following criteria:

1. Original studies, case reports, or case series describing CAWDs at any gestational age;
2. Prenatal or postnatal diagnosis confirmed by ultrasound, Doppler, and/or fetal MRI;
3. Reporting of diagnostic features, prenatal management, genetic evaluation, or perinatal outcomes;
4. Full-text availability in English;
5. Publication within the defined study period.

Studies were excluded if they met any of the following criteria:

1. Absence of patient-level clinical data;
2. Review articles, editorials, and conference abstracts without original cases;
3. Publications in languages other than English;
4. Duplicate publications describing the same patients;
5. Reports not consistent with true abdominal wall defects.

### 2.3. Study Selection and Data Extraction

All records retrieved from PubMed and Google Scholar were exported into a reference management software, and duplicate entries were removed. Titles and abstracts were screened for relevance to CAWDs, followed by full-text assessment according to the predefined inclusion and exclusion criteria. Reference lists of eligible articles were also manually reviewed to identify additional relevant studies.

Data extracted from the included publications comprised the type of congenital abdominal wall defect, gestational age at diagnosis, prenatal ultrasound and Doppler findings, fetal MRI findings when available, associated anomalies, genetic investigations, pregnancy outcome, delivery characteristics, and neonatal outcome. Owing to the heterogeneity of study designs and reported outcomes, a qualitative narrative synthesis was performed.

Because this review was designed as a narrative review rather than a systematic review, study selection, eligibility assessment, and data extraction were performed by a single author. Independent duplicate screening and data extraction were not undertaken.

### 2.4. Data Items and Outcomes

From each included publication, we extracted the following variables: type of congenital abdominal wall defect (gastroschisis, omphalocele, or BSA), maternal age, parity, relevant medical history, gestational age at diagnosis, prenatal ultrasound and Doppler findings, fetal MRI findings when available, associated structural anomalies, genetic testing results (karyotype, chromosomal microarray, or non-invasive prenatal testing), pregnancy management strategy, pregnancy outcome (continuation, termination of pregnancy, or intrauterine fetal demise), timing and mode of delivery, and neonatal outcome.

For gastroschisis, additional data regarding sonographic markers of disease severity (including bowel dilatation, bowel wall thickening, closed or vanishing gastroschisis, and fetal growth restriction) were recorded when reported. For omphalocele, the presence of liver herniation, associated anomalies, and genetic abnormalities was specifically documented. For BSA, the extent of body wall involvement, limb abnormalities, and placental or umbilical cord abnormalities were recorded.

Where individual studies lacked specific information for a given variable, that study was excluded from analyses of that particular variable, and missing data are indicated in the corresponding tables and text.

#### Risk of Bias and Level of Evidence 

Because the included evidence consisted predominantly of case reports, case series, retrospective cohorts, and narrative reviews, no formal risk-of-bias tool designed for randomized or comparative studies was applied. Instead, we assessed the completeness of reporting, including diagnostic confirmation, imaging documentation, genetic evaluation, follow-up duration, and clarity of pregnancy and neonatal outcomes. Studies with critically incomplete data were considered lower-quality evidence and were interpreted cautiously in the narrative synthesis.

### 2.5. Data Synthesis

Given the heterogeneity of the included studies with respect to study design, patient characteristics, types of congenital abdominal wall defects, prenatal imaging findings, associated anomalies, management strategies, and reported outcomes, a qualitative narrative synthesis was performed rather than a meta-analysis.

Findings were organized according to the type of congenital abdominal wall defect, associated anomalies, imaging modality, prenatal management, timing and mode of delivery, and perinatal outcomes. Where appropriate, descriptive comparisons were made across studies to highlight similarities and differences in clinical presentation, diagnostic features, prognostic factors, and management strategies.

### 2.6. Ethical Considerations

The study was conducted in accordance with institutional ethical standards. In addition to consent for study participation, written informed consent for the publication of clinical photographs and ultrasound images was specifically obtained from the legal guardians. All published images were fully anonymized, and no identifying patient information is included in the manuscript.

### 2.7. Study Limitations

The findings of this review should be interpreted in light of several limitations. First, the available evidence is predominantly derived from case reports and small case series, limiting the overall level of evidence and the generalizability of the conclusions.

Second, substantial heterogeneity existed across the included studies with respect to patient characteristics, types of CAWDs, prenatal imaging protocols, diagnostic criteria, management strategies, and outcome reporting, precluding quantitative synthesis and limiting direct comparisons between studies.

Furthermore, the lack of standardized prenatal ultrasound protocols and inconsistent reporting of imaging findings may have affected the comparability and reproducibility of the published data. The illustrative institutional case series also has inherent limitations, including its retrospective design, the small number of cases, and the absence of gastroschisis cases, which limits its ability to represent the full spectrum of congenital abdominal wall defects.

Finally, publication bias and incomplete reporting of clinical variables or follow-up outcomes may have influenced the available evidence, potentially under-representing uncomplicated or negative cases.

## 3. Illustrative Examples and Case Series

### 3.1. Illustrative Examples of Physiological Midgut Herniation

Physiological midgut herniation is a normal developmental event occurring between approximately 8 and 11 gestational weeks (g.w.) and represents an important differential diagnosis of first-trimester omphalocele. We present two representative first-trimester ultrasound examinations to illustrate its characteristic appearance and to emphasize the importance of avoiding false-positive diagnosis of CAWDs before completion of physiological bowel return.

*Example 1*.

A routine first-trimester ultrasound examination performed at 10 g.w. demonstrated physiological midgut herniation, which resolved completely on follow-up imaging.

*Example 2*.

**Figure 1:**
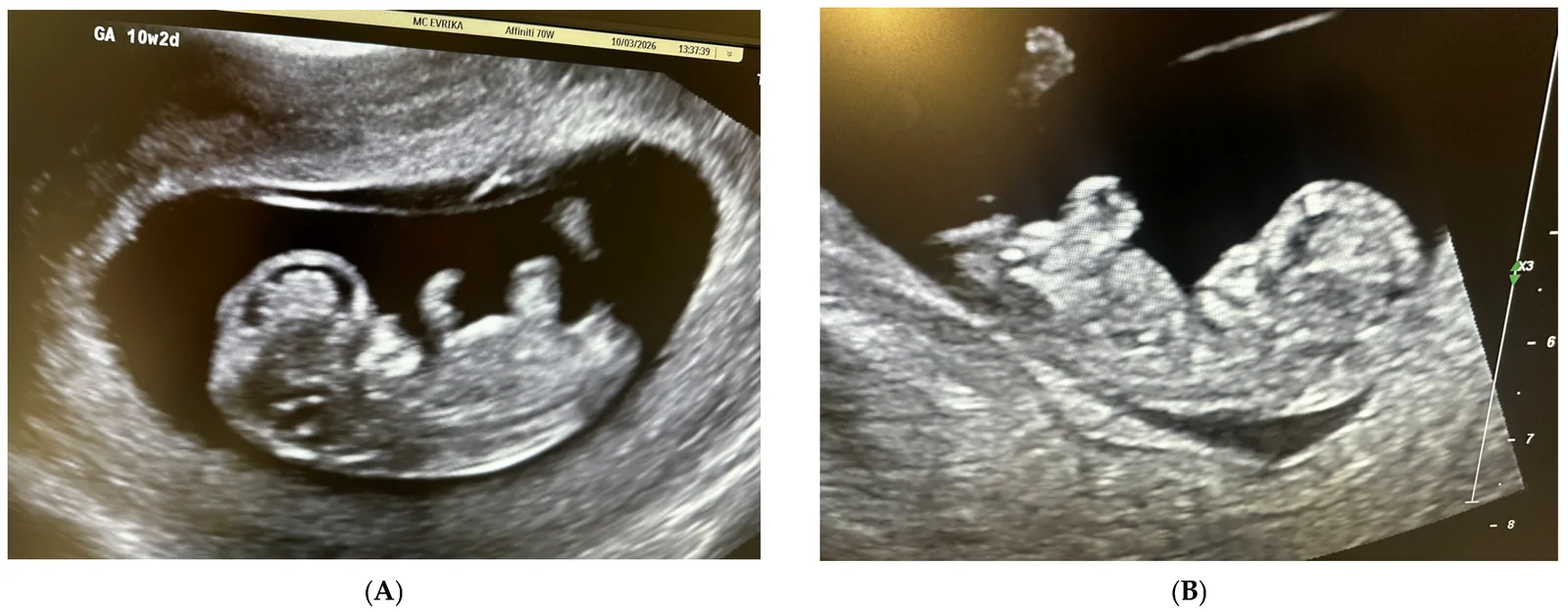
**Representative first-trimester ultrasound examples of physiological midgut herniation.** These images illustrate the normal transient herniation of the midgut into the base of the umbilical cord during early embryological development and highlight an important diagnostic pitfall in the differentiation from omphalocele before 11–12 weeks of gestation. (**A**) First-trimester sagittal ultrasound image of illustrative example 1 at 10 g.w. demonstrating normal physiological midgut herniation. A small midline protrusion at the base of the umbilical cord is visible, consistent with transient herniation of the midgut into the extraembryonic coelom. This finding is normal at this gestational age and should not be misinterpreted as omphalocele. (**B**) First-trimester sagittal ultrasound image of illustrative example 2 at 11 g.w. showing persistent but physiological midgut herniation, with no associated structural abnormalities. Follow-up confirmed normal embryological development with subsequent return of the bowel into the abdominal cavity.

A second first-trimester examination at 11 g.w. demonstrated the expected transient protrusion of the midgut into the base of the umbilical cord without associated structural abnormalities (Figure 1). Follow-up confirmed normal fetal anatomy.

### 3.2. Case 1: Omphalocele Associated with Trisomy 18

**Figure 2:**
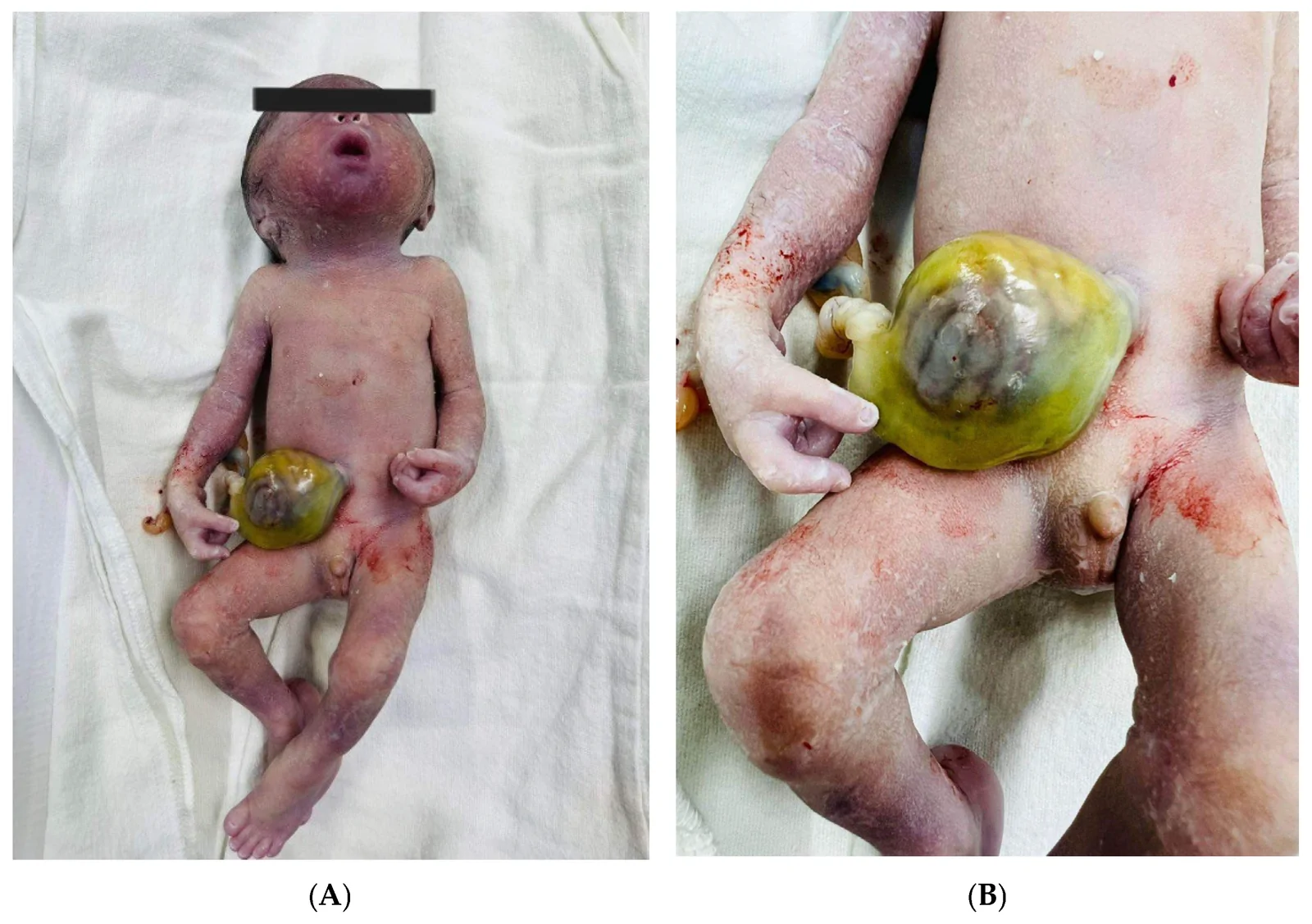
**Post-termination clinical photographs of omphalocele associated with trisomy 18.** (**A**) Whole-body view of a male fetus presenting with omphalocele. The characteristic translucent membranous sac covering the herniated abdominal viscera at the umbilical region is seen protruding from the abdomen. The sac appears tense, with areas of yellow-green discoloration. The surrounding skin is erythematous with patchy desquamation. The fetal limbs and external genitalia are grossly formed, and no obvious limb deformities are noted. Additional phenotypic features consistent with trisomy 18, including clenched hands with overlapping fingers, micrognathia and low-set ears, are visible. (**B**) Close-up view of the abdominal wall defect demonstrating a large omphalocele sac with visible internal contents, including intestinal loops. The membranous covering shows areas of thinning discoloration with vascular congestion. The base of the defect is located at the umbilical ring. Adjacent skin demonstrates erythema and superficial abrasions, providing detailed visualization of the lesion morphology.

A 20-year-old primigravida (G1P0) was referred following detection of multiple fetal abnormalities during the second-trimester morphology scan. Prenatal ultrasound demonstrated a midline omphalocele with a membranous sac containing herniated abdominal viscera, together with fetal growth restriction, clenched hands with overlapping fingers, and craniofacial abnormalities including micrognathia and low-set ears, findings highly suggestive of trisomy 18. Amniocentesis confirmed trisomy 18 (Edwards syndrome). After multidisciplinary counseling regarding the poor prognosis associated with omphalocele and chromosomal abnormalities, the pregnancy was medically terminated (Figure 2).

Prenatal ultrasound images were not available for inclusion because only post-termination clinical photographs had been archived. Nevertheless, the case illustrates the strong association between omphalocele and chromosomal abnormalities and underscores the importance of comprehensive genetic evaluation when omphalocele is identified prenatally.

### 3.3. Case 2: Omphalocele Associated with Intrauterine Fetal Demise

**Figure 3:**
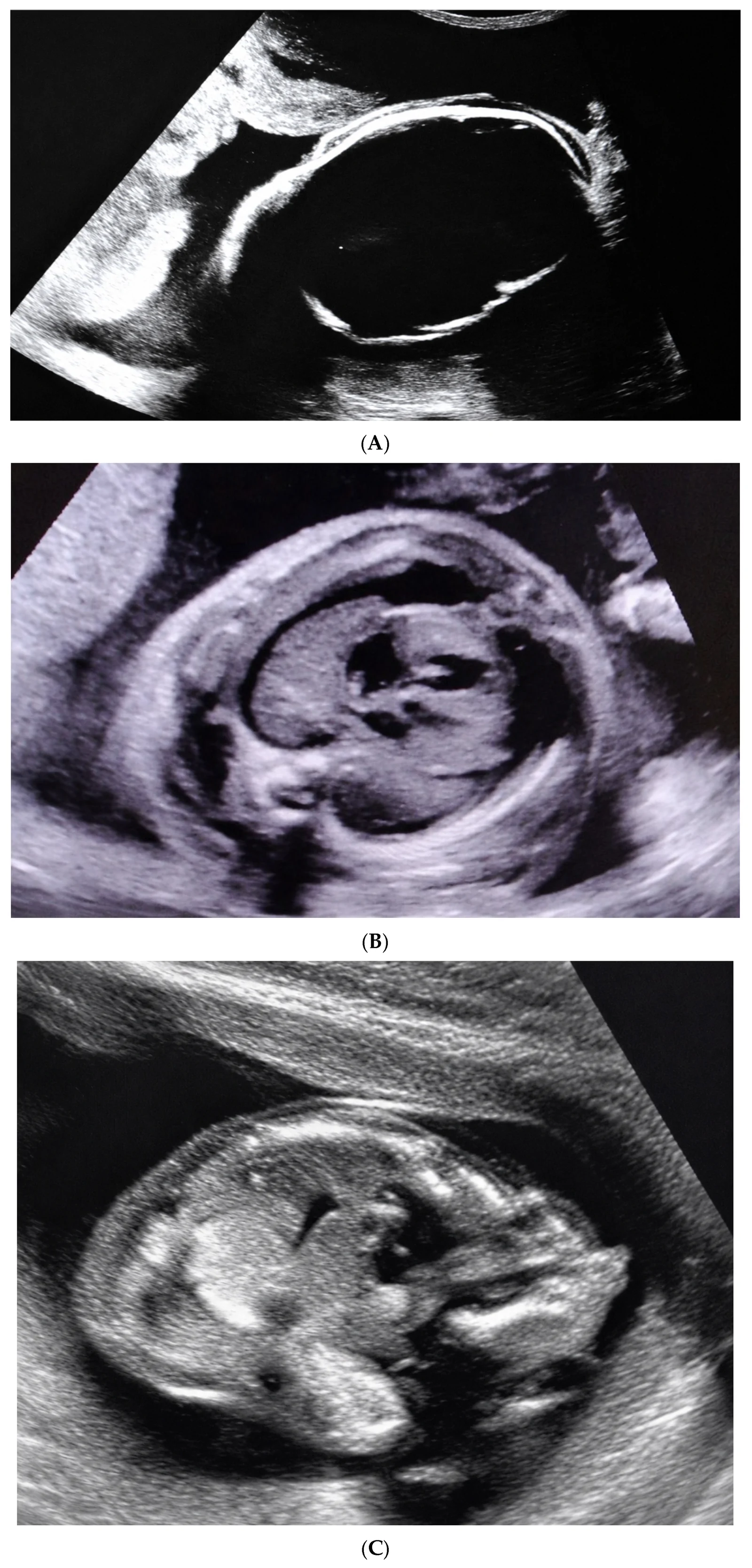
**Prenatal ultrasound findings in a case of intrauterine fetal demise associated with congenital abdominal wall defects.** (**A**) Ultrasound image illustrating features consistent with intrauterine fetal demise. Overlapping of the fetal cranial bones (Spalding sign) is evident, indicative of fetal death. (**B**) Ultrasound image depicting severe fetal hydrothorax. The transverse thoracic view demonstrates large bilateral anechoic pleural effusions with compression of the lungs and associated thoracic wall edema. No definite mediastinal shift is evident, as indicated by the symmetric distribution of the pleural effusions. The normal pulmonary architecture is poorly visualized because of the extensive fluid accumulation. (**C**) Ultrasound image revealing omphalocele. The axial view of the fetal abdomen shows a midline anterior abdominal wall defect at the base of the umbilical cord, with herniation of abdominal contents into a membrane-covered sac. The protruding mass appears well defined and contiguous with the umbilical cord insertion. The surrounding abdominal contour is disrupted, with externalized viscera enclosed within the sac.

A 25-year-old primigravida (G1P0) was referred following the diagnosis of intrauterine fetal demise, with estimated gestational age not precisely documented at the time of presentation. Maternal medical history was unremarkable. Prenatal ultrasound demonstrated a large midline omphalocele with herniation of abdominal viscera into a membrane-covered sac, associated with hydrothorax and the Spalding sign, confirming fetal demise (Figure 3).

Previous ultrasound examinations were not available for review. Due to the emergency clinical setting, fetal karyotyping and chromosomal microarray analysis were not performed. The presence of hydrops fetalis could not be reliably assessed based on the available imaging and documentation. No information regarding postmortem examination was available in the clinical records, limiting definitive evaluation for associated anomalies or syndromic conditions. Following multidisciplinary counseling, labor was induced, and the diagnosis was confirmed after delivery. The prenatal ultrasound examination was performed under emergency clinical circumstances following the diagnosis of intrauterine fetal demise. Consequently, only limited image documentation was archived, and higher-resolution ultrasound images were not available for inclusion in this article. The highest-quality available images have been included to illustrate the sonographic appearance of the defect despite this limitation.

### 3.4. Case 3: Body Stalk Anomaly

**Figure 4:**
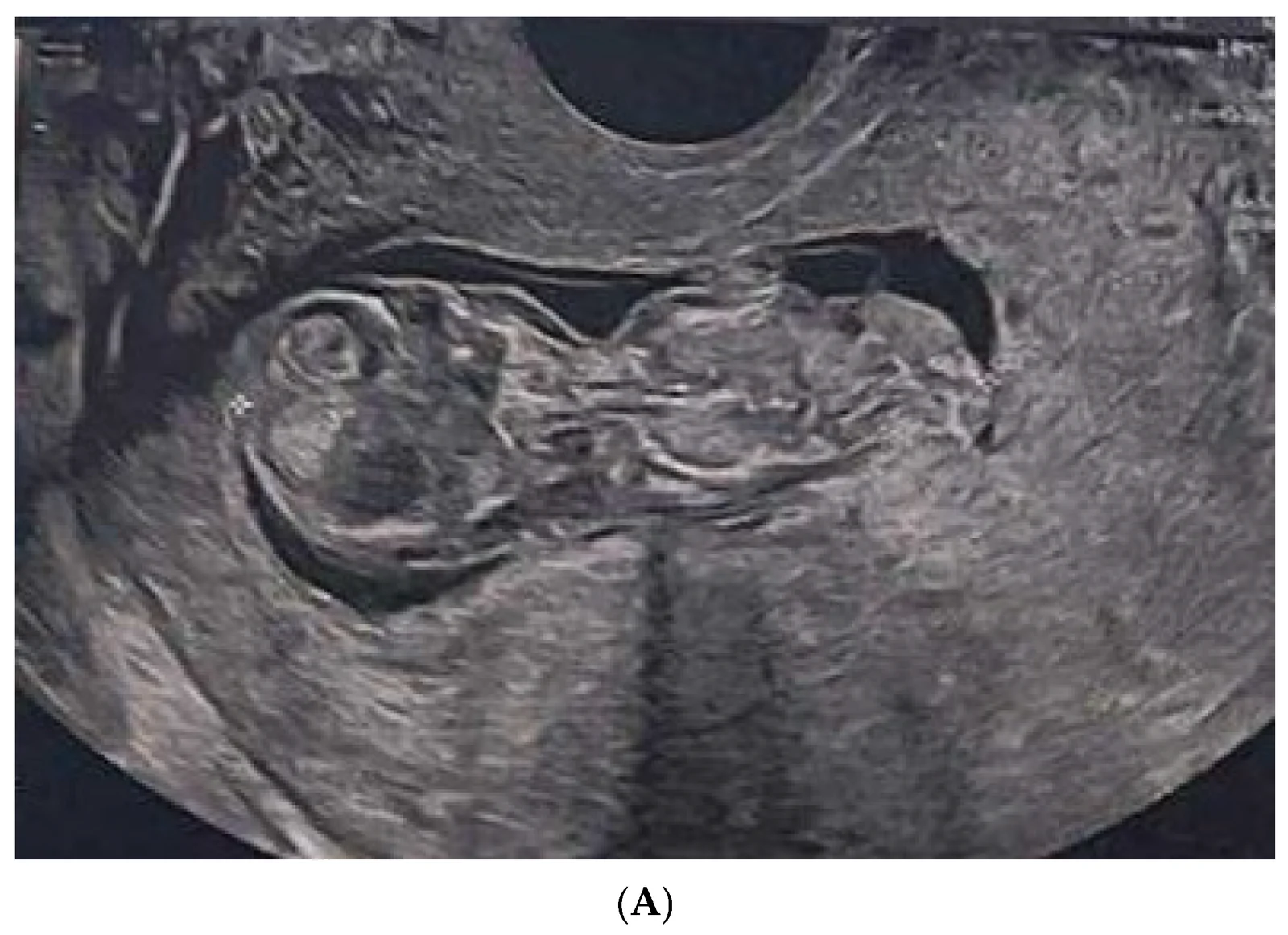

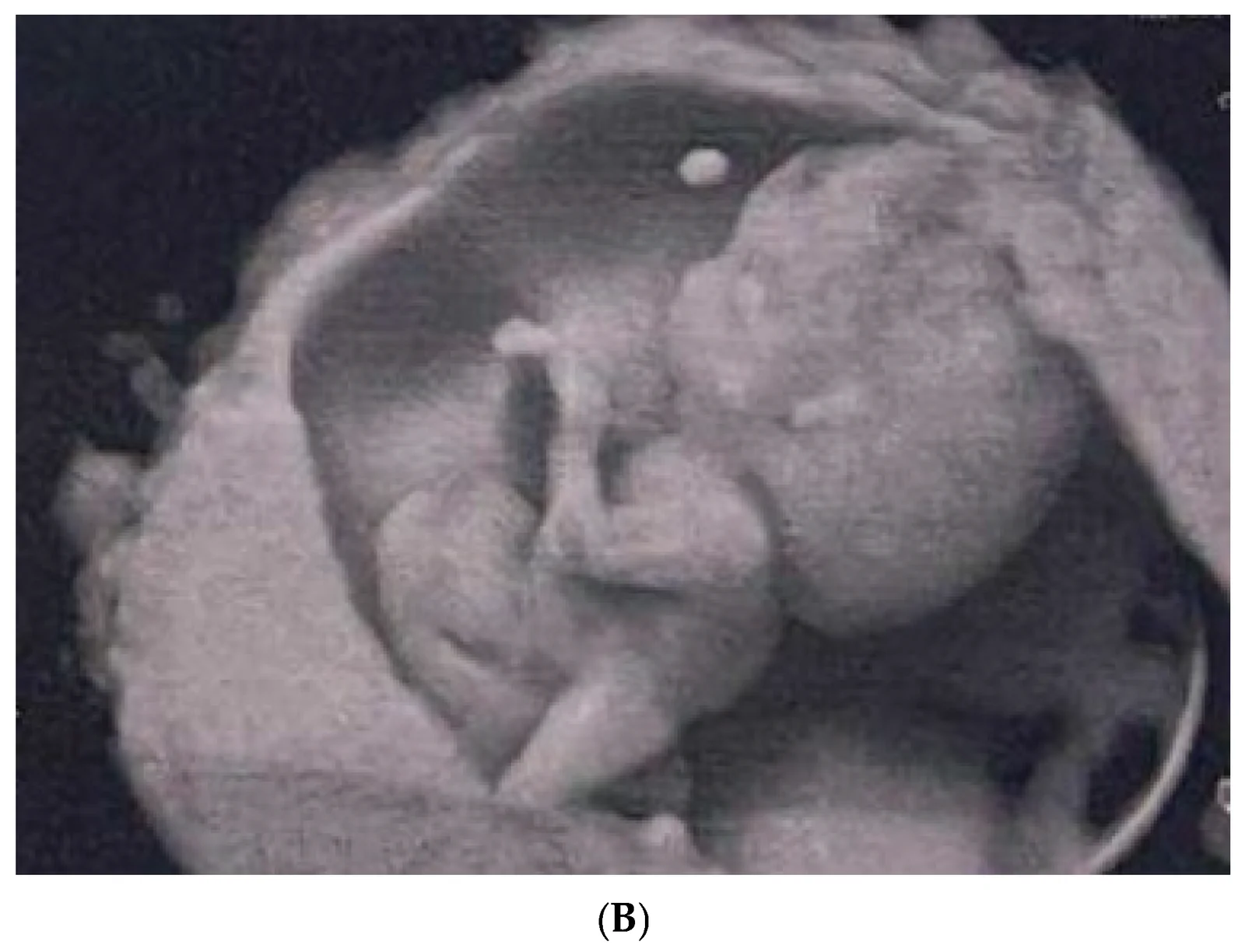
**First-trimester morphological assessment demonstrating body stalk anomaly.** First-trimester ultrasound documentation demonstrating fetal morphology with structural features consistent with body stalk anomaly. The composite panel presents multiple sectional views used for early anatomical assessments, highlighting the abnormal fetal body configuration and the relationship between the fetus, umbilical cord, and extraembryonic structures as recorded during routine imaging. (**A**) First-trimester ultrasound of BSA characterizing a large anterior abdominal wall defect with eviscerated abdominal contents, absent or severely shortened umbilical cord, and marked distortion of fetal anatomy including spine and thoracoabdominal structures. (**B**) First-trimester ultrasound of BSA exhibiting a large ventral body wall defect with eviscerated abdominal contents, absent or markedly shortened umbilical cord, close placental adherence, spinal curvature, and distortion of normal fetal anatomy.

A 30-year-old woman (G1P0), smoker with no other known medical conditions, was diagnosed on first fetal morphology scan with BSA (Figure 4). The pregnancy was terminated due to medical reasons. This case illustrates the severe end of the CAWD spectrum, characterized by early diagnosis and poor prognosis.

## 4. Results

The three pathological cases included in this series illustrate the broad clinical spectrum of CAWDs, ranging from isolated omphalocele to lethal body stalk anomaly.

In addition, two illustrative first-trimester ultrasound examples of physiological midgut herniation demonstrate an important normal developmental finding that should not be mistaken for omphalocele during early gestation.

Among pathological cases, one fetus was diagnosed with non-isolated omphalocele associated with trisomy 18 and multiple structural anomalies, underscoring the strong association between omphalocele and chromosomal abnormalities. Another case of omphalocele was identified in the context of intrauterine fetal demise, accompanied by hydrothorax and Spalding sign, highlighting the association between CAWDs and severe fetal compromise.

The most severe presentation was body stalk anomaly, diagnosed in the first trimester and characterized by extensive structural disruption and uniformly poor prognosis.

Across cases, prenatal ultrasound findings—including the presence of a covering membrane, liver herniation, associated anomalies, and overall structural integrity—were critical in distinguishing between isolated, complex, and lethal forms. These features informed clinical decision making regarding genetic testing, surveillance strategies, parental counseling, and pregnancy management.

Integration of case observations with current literature supports a structured, risk-adapted approach to prenatal diagnosis and perinatal management of CAWDs.

These findings support the concept of CAWDs as a spectrum of embryological and clinical severity, requiring individualized prenatal assessment and risk-adapted perinatal management.

The main characteristics of the three pathological cases included in this case series are summarized in Table 1. *Representative examples of physiological midgut herniation are presented separately (Figure 1) because they represent normal embryological findings rather than CAWDs*.

## 5. Discussion

### 5.1. Epidemiology

The following section reviews current epidemiologic trends and risk factors that shape contemporary approaches to diagnosis and management strategies.

#### 5.1.1. Incidence and Prevalence

CAWDs are predominantly represented by gastroschisis and omphalocele, and, less commonly, BSA and LBWC. Gastroschisis has historically been reported in approximately 1 in 4000 live births in large population cohorts [4].

Recent multinational surveillance data indicate a gastroschisis prevalence of approximately 3.06 per 10,000 total births, with regional heterogeneity (e.g., Europe~1.49/10,000; Latin America~3.80/10,000; North America~4.32/10,000) [5,6].

**Figure 5:**
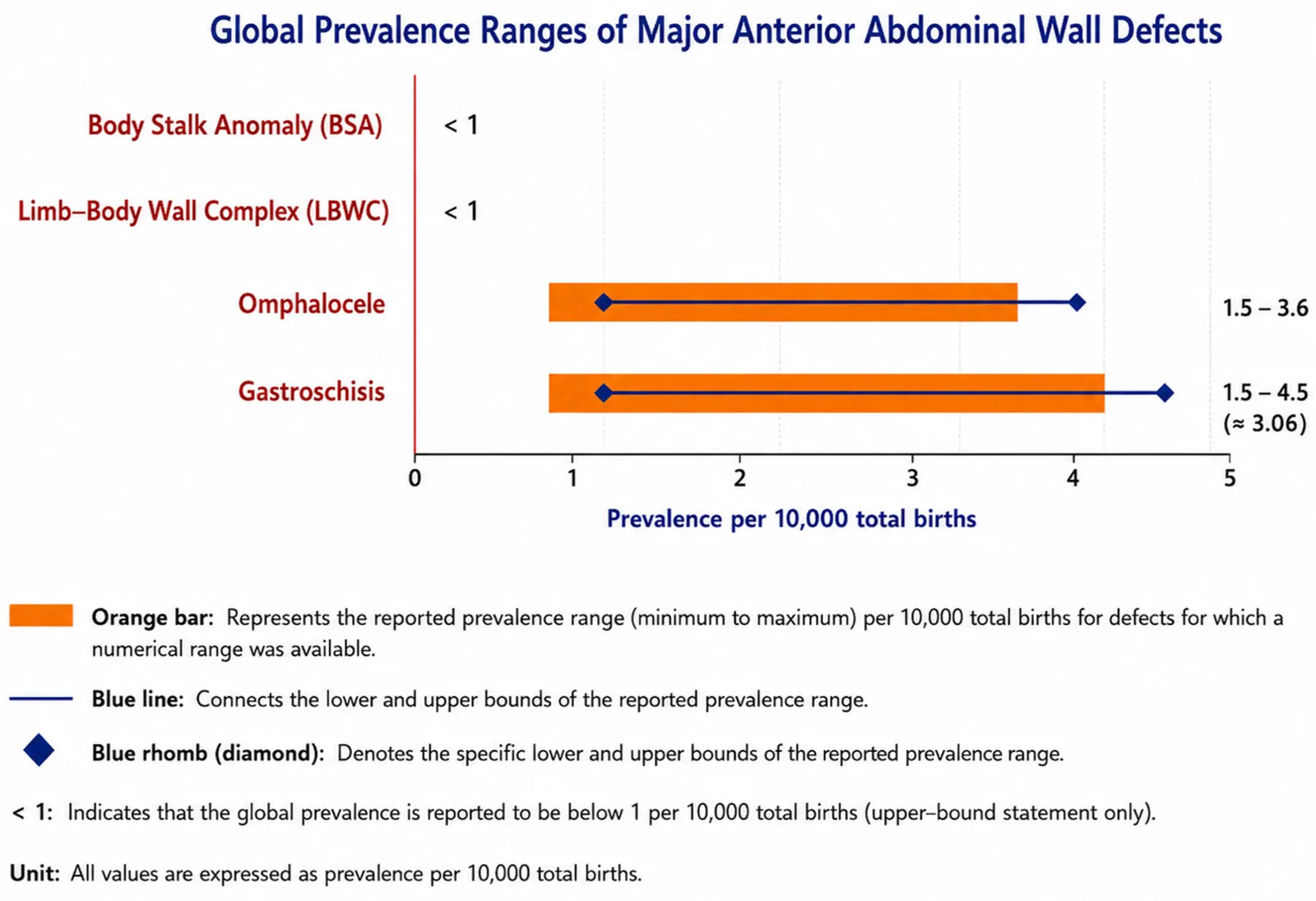
**Global prevalence ranges of major anterior abdominal wall defects.** Comparative prevalence of gastroschisis, omphalocele, BSA, and LBWC highlighting the markedly lower frequency of BSA and LBWC relative to isolated abdominal wall defects. **Note.** Prevalence values are expressed per 10,000 total births. Gastroschisis: approximately 3.06 per 10,000 total births; reported range 1.5 to 4.5 per 10,000 total births. Omphalocele: reported range 1.5 to 3.6 per 10,000 total births. BSA and LBWC: reported prevalence below 1 per 10,000 total births.

Pooled meta-analyses confirm that global gastroschisis prevalence generally ranges from 1.5 to 4.5 per 10,000 births, and omphalocele prevalence from 1.5 to 3.6 per 10,000 births [7,8] (Figure 5). Notably, increased detection rates in early gestation (9–11 g.w.) are primarily related to physiological midgut herniation, a normal developmental process of the first trimester that typically resolves by approximately 12 g.w., and may mimic omphalocele on early ultrasound, leading to a subsequent decrease in detectable cases [9,10].

Gastroschisis prevalence is similar between males and females, while higher prevalence has been reported among singleton pregnancies and among mothers < 20 years of age; differences have also been reported across racial and ethnic groups [5,11].

BSA and LBWC are rare, severe defects, typically resulting in fetal or early neonatal loss, with reported prevalence estimates below 1 per 10,000 births in population-based registries [12,13].

#### 5.1.2. Temporal Trends

Longitudinal surveillance data demonstrate subastantial temporal variation in gastroschisis prevalence, including a marked increase from the late 1990s to the late 2000s followed by a subsequent decline, with the highest prevalence observed among younger maternal age groups [5,14].

However, recent national datasets suggest this upward trend may be plateauing or even declining in some high-income settings, potentially reflecting shifts in maternal demographics and modifiable exposures [6,15].

By contrast, omphalocele prevalence has shown considerable temporal and geographical variation, with differences in reported estimates partly influenced by prenatal detection and termination practices [8].

Due to their rarity and frequent prenatal loss, trends in BSA and LBWC are difficult to quantify; available population-based data suggest low and relatively stable prevalence, particularly for LBWC [12,16].

#### 5.1.3. Geographic Distribution

Gastroschisis demonstrates geographic heterogeneity, with higher prevalence in the Americas and lower reported rates in some European regions, although data from low- and middle-income countries remain limited [5,6].

Socioeconomic factors, maternal nutrition, and environmental exposures have been proposed as contributors to regional differences, although the relative contributions of these factors remain uncertain [17,18].

Omphalocele also shows geographic variation in reported birth prevalence, with differences potentially influenced by variations in detection and reporting practices. [8].

BSA, while rare globally, has been described in multiple regions with similar low prevalence, reflecting their sporadic, severe nature rather than true regional differences in risk [12,13].

#### 5.1.4. Maternal Risk Factors

A robust epidemiological pattern is the strong association of gastroschisis with young maternal age, particularly mothers under 20 years [5,6,11]. Additional maternal risk factors for gastroschisis include low pre-pregnancy body mass index (BMI), maternal smoking, and other lifestyle exposures (including illicit drug use and alcohol consumption), although effect sizes vary across populations [19,20].

In contrast, omphalocele is more prevalent among mothers of advanced reproductive age (typically >40 years) and is often accompanied by other major anomalies [7]. In addition, maternal disorders of glycemic control, including pregestational and gestational diabetes, have also been associated with an increased risk of congenital anomalies, including omphalocele [21].

There are no well-established maternal risk factor associations specific to BSA beyond general sporadic embryopathy and chromosomal anomalies; many cases are identified early in gestation due to severe structural disruption [12,22].

#### 5.1.5. Chromosomal and Syndromic Associations

A key epidemiological distinction is the association with chromosomal abnormalities. Omphalocele is strongly linked to chromosomal anomalies such as trisomy 13, trisomy 18, and trisomy 21 and may occur in syndromic conditions (e.g., Beckwith–Wiedemann spectrum and pentalogy of Cantrell) [23].

Gastroschisis, in contrast, is most often an isolated defect, with chromosomal abnormalities being rare [4,6,14].

BSA is frequently associated with serious neural tube defects, limb abnormalities, and amniotic constriction bands and may coincide with chromosomal abnormalities in a minority of cases when detailed genetic testing is performed [22].

A summary of the main epidemiological characteristics of the principal congenital abdominal wall defects is provided in Table 2. 

### 5.2. Embryological Continuum and Phenotypic Severity

Normal formation of the anterior abdominal wall occurs between the 4th and 6th g.w. and depends on coordinated cephalic, caudal, and lateral folding of the trilaminar embryonic disc [24]. During this process, the lateral body folds migrate ventrally and fuse at the midline, enclosing the abdominal viscera within the peritoneal cavity, while the lateral mesoderm and ectoderm form the primary abdominal wall [25,26,27]. Simultaneously, the yolk sac narrows and integrates into the embryo to form the primitive gut, and the connecting stalk elongates to become the umbilical cord [25,26,28].

The timing, extent, and mechanism of disruption determine the clinical presentation of abdominal wall defects [29].

**Figure 6:**
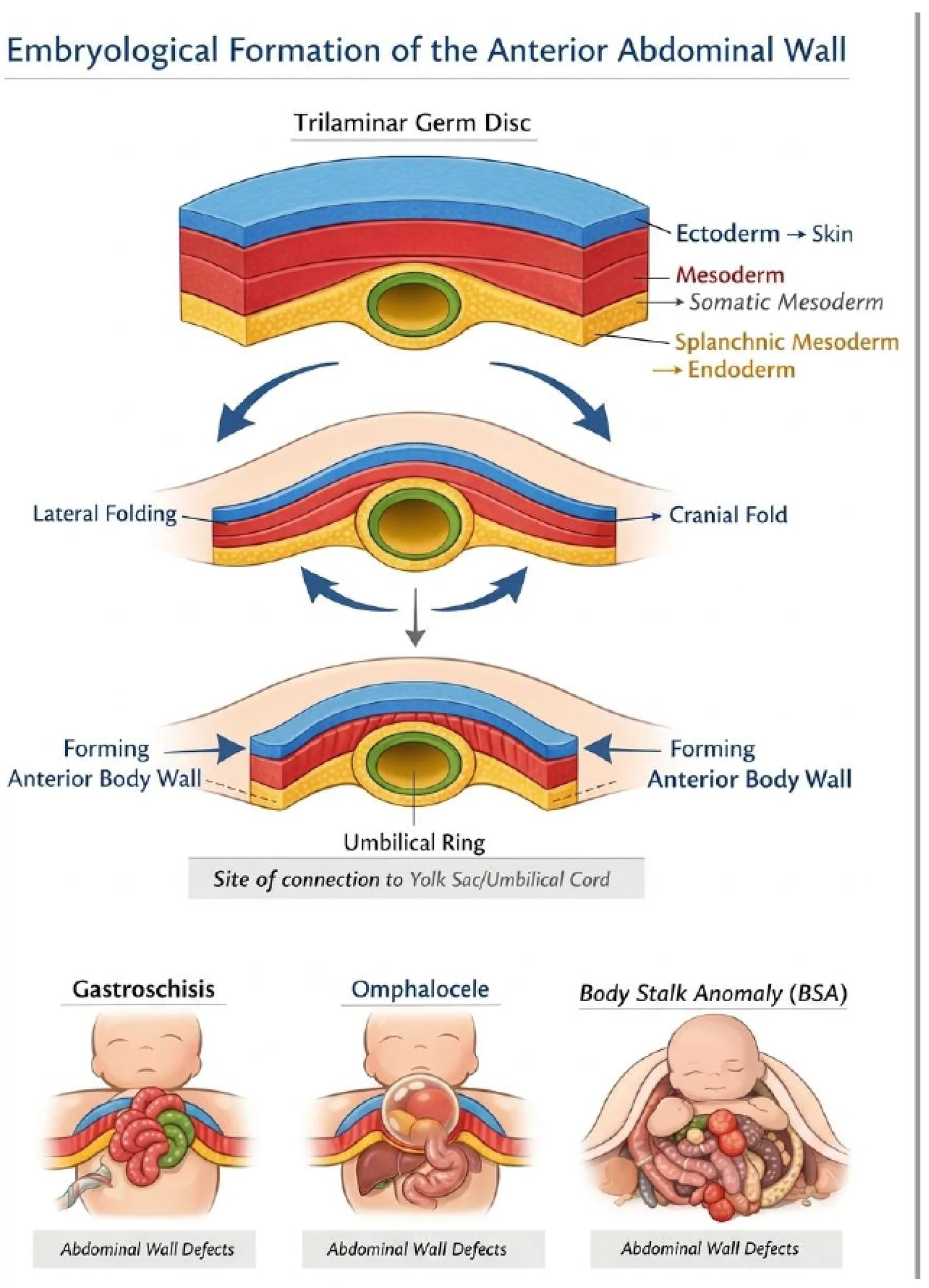
**Embryological basis of anterior abdominal wall development and congenital abdominal wall defects**.

Figure 6 summarizes the normal development of the anterior abdominal wall and the defects that arise when this process is disrupted. During embryogenesis, coordinated cranial and lateral folding of the trilaminar germ disc brings the somatic mesoderm and ectoderm to the midline, forming the ventral body wall and enclosing the abdominal organs. The umbilical ring represents the transient connection between the embryo and the yolk sac. Abnormal folding or incomplete midline closure results in distinct congenital anomalies.

From an embryological perspective, these defects represent a spectrum that corresponds to the degree of disturbance in normal body wall morphogenesis. Broadly, they may be classified into isolated defects, such as gastroschisis and omphalocele, complex midline malformation syndromes (e.g., pentalogy of Cantrell), and severe cloacal or caudal developmental anomalies (e.g., cloacal exstrophy or body stalk anomaly), according to the timing and extent of morphogenic disturbance.

Isolated defects arise from localized failure of ventral wall closure and typically involve limited perturbation of other organ systems, contributing to their comparatively favorable postnatal survival profiles.

In contrast, more severe defects—including pentalogy of Cantrell and cloacal exstrophy—result from broader disruption in embryonic morphogenesis affecting multiple structures, including the musculoskeletal, cardiovascular, urogenital, and central nervous systems, and are therefore associated with significantly poorer prognosis [12,30,31]. The clinical spectrum of CAWDs thus mirrors the underlying embryologic severity of the developmental insult.

Within this continuum, BSA, or LBWC, represents one of the most extreme and uniformly lethal forms of ventral wall disruption. BSA is characterized by a large thoracoabdominal wall defect, severe spinal deformities, absent or markedly shortened umbilical cord, and frequent limb and visceral anomalies. Unlike isolated abdominal wall defects, which reflect a focal failure of closure, BSA arises from a fundamental failure of early embryonic folding and is incompatible with postnatal survival [32,33].

Early antenatal ultrasound plays a central role in distinguishing these anomalies, as timely identification directly influences prenatal surveillance, parental counseling, and perinatal decision making. Understanding these anomalies within an embryological continuum strengthens this diagnostic process: defects arising from localized failure of ventral wall closure generally present as isolated conditions with favorable surgical outcomes, whereas those resulting from global disruption of embryonic folding, such as BSA, are associated with multisystem involvement and uniformly lethal prognosis. Conceptualizing CAWDs along this developmental spectrum, with BSA representing the most severe end, provides a unified framework that enhances prognostic accuracy and supports individualized management strategies.

While this embryological framework provides a broad understanding of CAWDs, further stratification within individual anomalies reveals additional clinical and pathophysiological heterogeneity with direct implications for prenatal surveillance and perinatal management.

#### 5.2.1. Gastroschisis: Spectrum and Clinical Stratification

Gastroschisis typically presents as a small paraumbilical abdominal wall defect, measuring approximately 2–3 cm, and is thought to result from involution of the right umbilical vein. Through this defect, the midgut herniates freely into the amniotic cavity. Direct exposure of the bowel to amniotic fluid results in inflammatory changes, including bowel wall thickening, edema, and the formation of an inflammatory peel. In contrast, bowel shortening is primarily associated with vascular compromise and intestinal injury, particularly in complex gastroschisis, where ischemia, atresia, or bowel necrosis may occur.

Evidence suggests that thrombosis of the right umbilical vein can occur even when the vessel is structurally normal [34] and that early involution of the right omphalomesenteric artery is frequently associated with this defect [35,36]. Such vascular compromises likely diminish oxygen and nutrient delivery to the developing tissues, disrupting normal trophic support. This contributes to malformations of ectodermal and mesodermal structures, as well as abnormal formation of the terminal portion of the omphalomesenteric artery supplying the right mesogastric region [37].

These developmental variations underlie the broad clinical spectrum observed in gastroschisis. The condition should be understood not as a single entity but as a continuum encompassing both severity-based and progressive forms. Recent reviews emphasize the importance of prenatal classification of gastroschisis into simple and complex forms, which influences perinatal management decisions and informs long-term prognosis [37]. This framework provides the basis for the most widely adopted clinical classification. 

Clinically, simple gastroschisis is characterized by intact bowel without complications, while complex gastroschisis involves intestinal atresia, necrosis, perforation, or volvulus, all of which significantly increase morbidity and mortality, reducing survival rate from >90% to 70–80% in developed countries [38]. Complex gastroschisis occurs in approximately 11–28% of patients, with intestinal atresia being the most common associated anomaly, affecting about 10–15% of cases [38].

**Figure 7:**
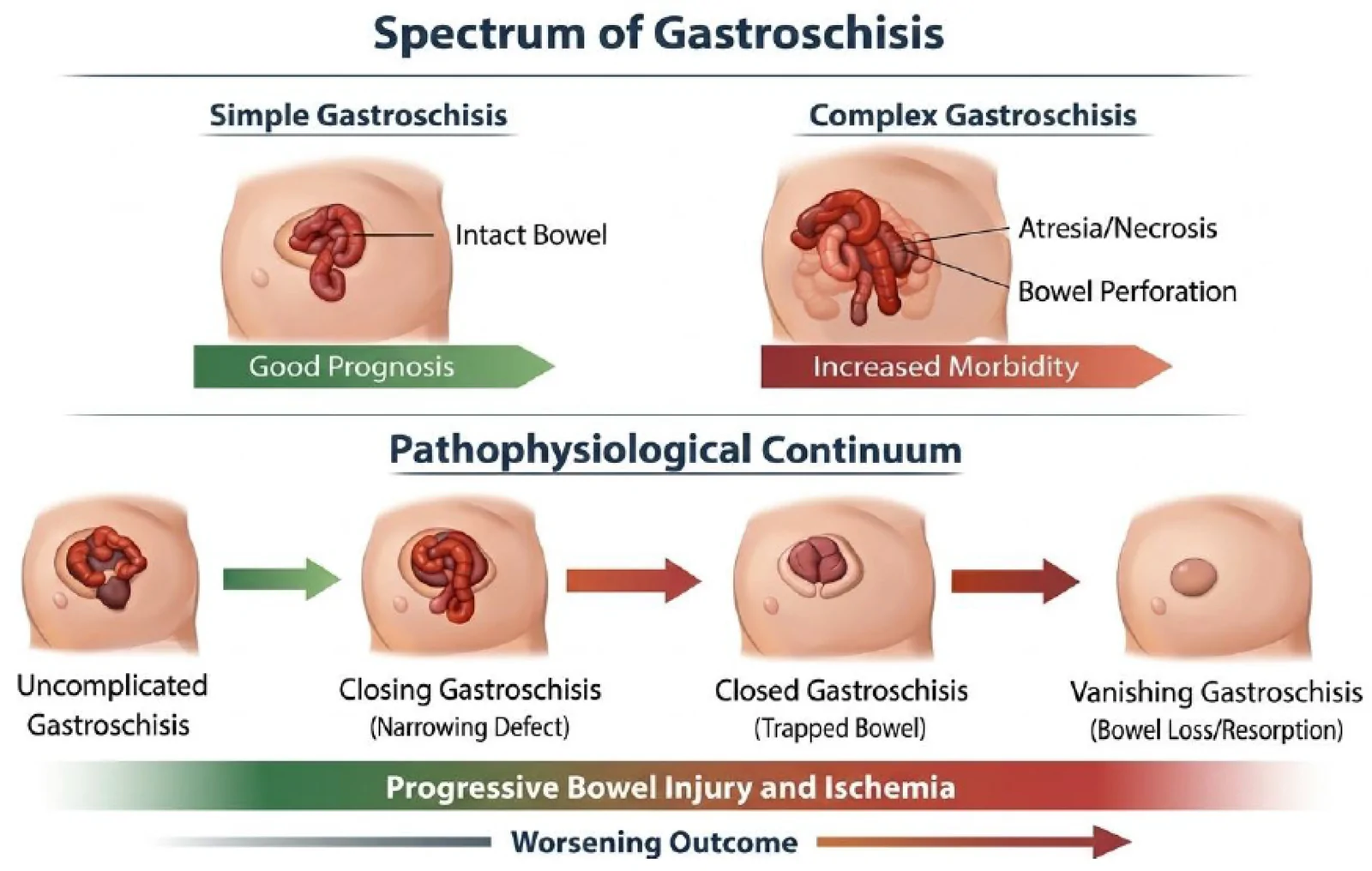
**Spectrum of gastroschisis**.

Building on this classification, a pathophysiological continuum has been described, ranging from uncomplicated gastroschisis to closing, closed, and vanishing gastroschisis (Figure 7). In these progressive variants, constriction of the abdominal wall defect can lead to vascular compromise, bowel ischemia, and, in severe cases, intestinal loss [39].

- Closing gastroschisis occurs when the abdominal wall defect progressively shrinks during the last trimester, potentially compressing the herniated viscera and causing bowel obstruction and/or mesenteric ischemia [39].
- Closed gastroschisis refers to the rare scenario in which the abdominal wall is completely closed at birth, sometimes leaving only rudimentary herniated viscera bowel [40].
- Vanishing gastroschisis represents the most severe outcome, with complete loss of the herniated bowel, often accompanied by secondary intestinal atresia [41].

The upper panel depicts the clinical classification of gastroschisis, distinguishing simple gastroschisis (intact bowel with good prognosis) from complex gastroschisis (presence of intestinal atresia, necrosis, or perforation, associated with increased morbidity and lower survival rates).

The lower panel illustrates the pathophysiological continuum of gastroschisis, demonstrating the dynamic in utero progression from uncomplicated gastroschisis to closing gastroschisis (narrowing abdominal wall defect), closed gastroschisis (trapped bowel), and vanishing gastroschisis (complete bowel loss or resorption). Progressive constriction of the abdominal wall may lead to vascular compromise, bowel ischemia, obstruction, or secondary atresia.

Therefore, it is crucial to detect signs of abdominal wall closure in utero to optimize perinatal management and anticipate potential complications. Prenatal ultrasound plays a pivotal role in identifying progression along this spectrum, with findings such as increasing intra-abdominal bowel dilation, thickened bowel loops, disappearance of extra-abdominal bowel, and fetal growth restriction serving as early indicators of worsening disease [42,43].

Recognition of gastroschisis as both a clinical severity spectrum (simple vs. complex) and a dynamic in utero process is essential for accurate risk stratification, parental counseling, and optimization of perinatal management strategies [43].

#### 5.2.2. Omphalocele: Spectrum and Clinical Stratification

In contrast to gastroschisis, omphalocele represents a distinct entity with major differences in embryological origin, epidemiology, associated anomalies, and overall prognosis. These differences are clinically significant, particularly given the markedly higher prevalence of associated structural and chromosomal abnormalities in omphalocele, which constitute the principal determinants of morbidity and mortality [23,44,45,46].

From an embryological perspective, omphalocele arises from abnormal development of the ventral abdominal wall during early gestation. Under normal conditions, lateral body folds migrate and fuse in the midline during the 3rd and 4th g.w., forming the anterior abdominal wall. Disruption of this process results in a persistent midline defect. In addition, physiological midgut herniation, which occurs between 8 and 10 g.w. and normally resolves by 10–12 weeks following a 270° counterclockwise rotation, may fail to return to the abdominal cavity [47].

Several theories have been proposed to explain the pathogenesis of omphalocele. The classical hypothesis attributes the defect to failure of the herniated midgut to return to the abdominal cavity. However, this mechanism alone does not adequately explain larger defects containing solid organs such as the liver. Consequently, a more comprehensive and widely accepted theory suggests that primary failure of lateral body fold closure during early embryogenesis results in a larger ventral wall defect, permitting herniation of multiple intra-abdominal organs [46,48]. Recent genetic and molecular studies further support a multifactorial etiology involving multiple developmental pathways and embryological stages, rather than a single isolated defect [46].

Morphologically, omphalocele is characterized by a midline abdominal wall defect at the base of the umbilical cord, with herniated viscera enclosed within a membranous sac composed of three layers: an inner peritoneal layer, an intermediate layer of Wharton’s jelly, and an outer amniotic layer [45,47]. The umbilical cord inserts at the apex of the sac, which represents a key distinguishing feature from gastroschisis. Importantly, the presence of this covering membrane protects the herniated contents from direct exposure to amniotic fluid, thereby reducing inflammatory bowel damage.

Omphalocele exhibits considerable phenotypic variability and is commonly classified according to defect size and content. Smaller defects typically contain bowel loops only, whereas larger or “giant” omphaloceles frequently include the liver and other solid organs. This distinction has important prognostic implications, as liver-containing omphaloceles are associated with increased risk of pulmonary hypoplasia and adverse neonatal outcomes [44].

A defining characteristic of omphalocele is its strong association with additional anomalies, reported in up to 50–80% of cases in recent series [23,45]. These include structural malformations (most commonly congenital heart defects) as well as chromosomal abnormalities, particularly trisomies 13 and 18, and syndromic conditions such as Beckwith–Wiedemann syndrome [23,45]. Contemporary evidence emphasizes the importance of distinguishing isolated from non-isolated omphalocele, as isolated cases are associated with significantly improved survival and long-term outcomes [23].

In contrast to gastroschisis, where associated anomalies are relatively uncommon, the high burden of concomitant abnormalities in omphalocele largely determines prognosis. Therefore, accurate prenatal differentiation between these two entities is essential for appropriate parental counseling, genetic evaluation, and perinatal management.

Given this marked heterogeneity in anatomical presentation, associated anomalies, and clinical outcomes, a structured classification of omphalocele is essential for accurate risk stratification and management.

##### Classification of Omphalocele

**Figure 8:**
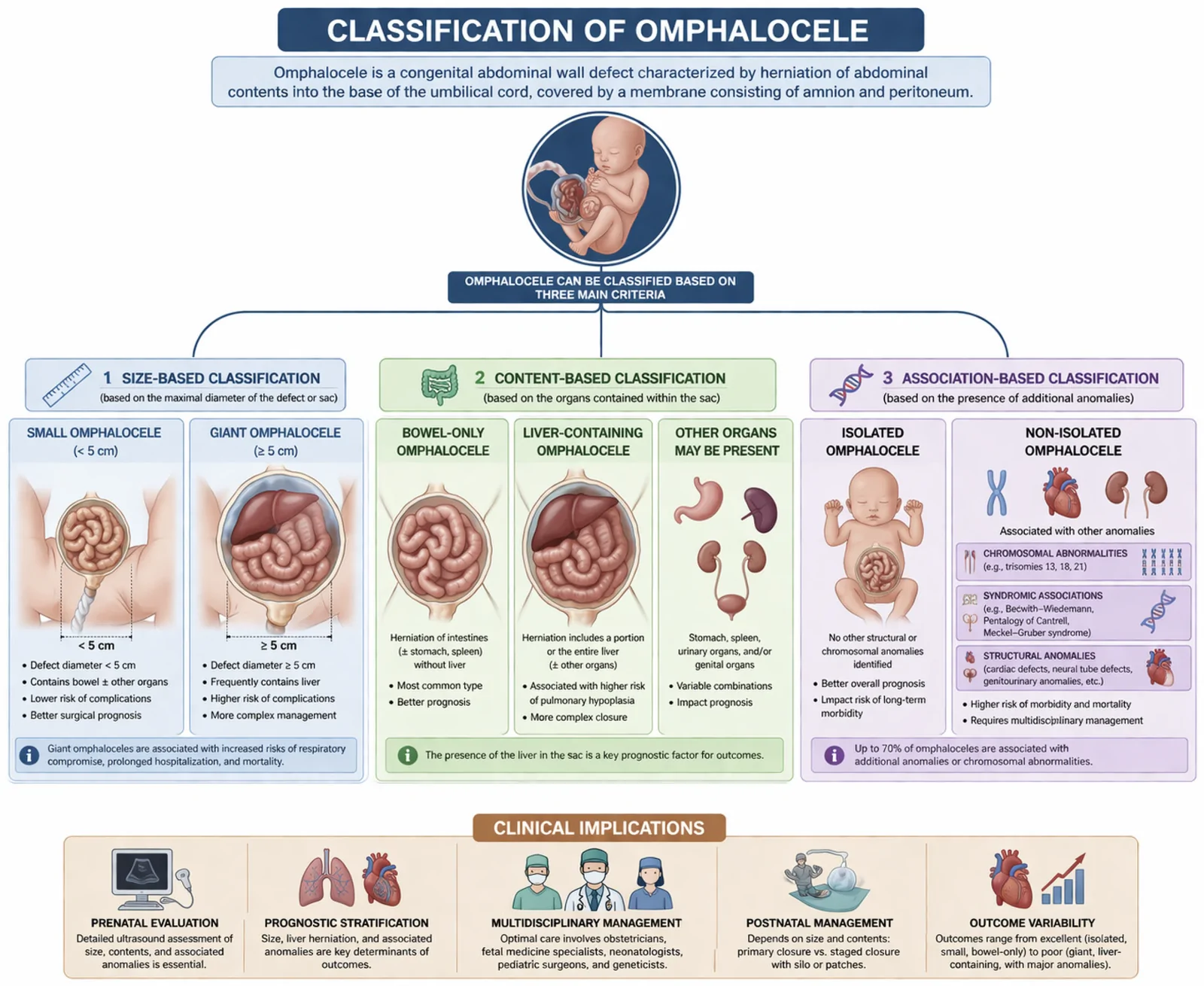
**Omphalocele classification. Size:** small < 5 cm/giant ≥ 5 cm. **Content:** bowel-only/liver ± other organs. **Associated anomalies:** isolated/non-isolated. These criteria guide prenatal risk assessment and postnatal management.

The main classification systems for omphalocele, based on anatomical, clinical, and prognostic parameters, are summarized in Table 3 and illustrated in Figure 8.

Among the proposed classification systems, the distinction based on associated anomalies represents the most clinically relevant parameter, as it directly correlates with survival and long-term outcomes. Isolated omphalocele is generally associated with a favorable prognosis, whereas non-isolated forms, characterized by structural or chromosomal abnormalities, constitute the major determinant of morbidity and mortality [49,50].

The size- and content-based classification further refines risk stratification. In particular, giant omphaloceles, defined by the presence of the liver within the hernia sac, are associated with reduced abdominal domain and an increased risk of pulmonary hypoplasia, often requiring staged or complex surgical repair [44,51]. In contrast, smaller defects containing bowel loops only are typically associated with more favorable outcomes [36].

Finally, although less frequently emphasized, the integrity of the covering sac may influence perinatal management. An intact sac provides mechanical and biochemical protection of the herniated viscera, whereas rupture—either prenatally or during delivery—can expose abdominal contents to external factors, increasing the risk of infection and necessitating urgent intervention [52,53].

#### 5.2.3. Limb–Body Wall Complex/Body Stalk Anomaly: Phenotypic Spectrum and Prenatal Classification

BSA, frequently considered within the LBWC spectrum, represents the most severe end of CAWDs and results from profound disruption of early embryonic folding. Unlike isolated abdominal wall defects, LBWC/BSA is characterized by a major thoracoabdominal wall disruption, severe spinal deformities, absent or markedly shortened umbilical cord, and multisystem malformations incompatible with postnatal survival. Diagnostics definitions vary; however, the criteria proposed by Van Allen et al., requiring two to three major findings—thoracoabdominal wall defects with visceral herniation, limb abnormalities, and craniofacial defects—remain among the most widely cited diagnostic frameworks.

Recent retrospective evidence evaluating 37 fetuses with LBWC further emphasized the feasibility and reliability of first-trimester diagnosis and identified consistent prenatal ultrasound features, including severe ventral wall defects, placental adherence, spinal abnormalities, limb defects, and umbilical cord anomalies, reinforcing the importance of systematic prenatal assessment and early counseling [54].

Phenotypically, LBWC has been subclassified into placental cranial and placental abdominal phenotypes. The placental cranial phenotype is more frequently associated with craniofacial defects, neural tube abnormalities, and amniotic band-related findings, whereas the placental abdominal phenotype is characterized predominantly by extensive thoracoabdominal wall defects, visceral evisceration, severe scoliosis, and urogenital abnormalities. Although these classifications have limited implications for survival, given the uniformly poor prognosis of LBWC/BSA, they may improve prenatal recognition, differential diagnosis, and phenotypic characterization. Within the proposed continuum of CAWDs, LBWC/BSA represents the extreme end of developmental disruption, emphasizing the importance of early recognition for accurate counselling and individualized pregnancy management.

### 5.3. Clinical Implications and Prenatal Management of CAWDs

- Prenatal Diagnosis

From a clinical perspective, fetal abdominal wall defects are typically identified during routine first- or early second-trimester ultrasound screening. Diagnosis relies on high-resolution ultrasonography, enabling detection as early as the late first trimester in specialized settings and consistently at the mid-trimester anatomy scan [31,32,33]. In addition to conventional grayscale imaging, real-time ultrasonography permits dynamic assessment of fetal bowel motility, while color and pulsed Doppler ultrasound provide evaluation of vascular perfusion of the herniated viscera and umbilical vessels, particularly when bowel compromise or associated vascular abnormalities are suspected. Precise prenatal characterization requires systematic evaluation of defect location and dimensions, its relationship to the umbilical cord, the presence or absence of a covering membrane, and associated structural anomalies. On this basis, the recommended work-up includes (1) comprehensive anatomic survey; (2) targeted assessment of cord insertion, liver herniation, and spinal alignment; (3) fetal echocardiography; and (4) genetic evaluation via chorionic villus sampling or amniocentesis when indicated.

#### 5.3.1. Role of Fetal MRI

Fetal magnetic resonance imaging (MRI) serves as a complementary second-line imaging modality for the evaluation of CAWDs, particularly in cases where ultrasonographic findings are inconclusive or when additional anatomical characterization is required. While ultrasound remains the primary screening tool, fetal MRI provides superior soft-tissue contrast and a larger field of view, enabling more accurate differentiation of CAWD subtypes and comprehensive assessment of associated anomalies that may influence prognosis and perinatal management [55,56,57].

**1.**  **Accurate differentiation of CAWD types**

Fetal MRI improves the characterization and differentiation of CAWDs by providing multiplanar anatomical visualization of the abdominal wall defect and herniated contents, thereby supporting distinction between gastroschisis and omphalocele when ultrasound findings are equivocal [56,57].

**2.**  **Superior evaluation of exposed organs**

Fetal MRI allows for detailed assessment of herniated viscera with improved soft-tissue resolution, facilitating detection of bowel complications such as obstruction, atresia, distension, or ischemic changes. It also enables more accurate evaluation of the volume and composition of herniated organs, particularly the liver and bowel, which are relevant for prognostic assessment and postnatal surgical planning [55,57].

**3.**  **Detection of co-occurring syndromes and structural anomalies**

Fetal MRI provides whole-fetus evaluation and is particularly valuable in identifying associated anomalies, especially in omphalocele, which is frequently linked to chromosomal and syndromic conditions such as trisomies 13, 18, and 21 and Beckwith–Wiedemann syndrome. It allows for the assessment of additional structural abnormalities involving the central nervous system, thorax, heart, and skeletal system, including thoracoabdominal disproportion and pulmonary hypoplasia, both of which significantly influence neonatal survival and prognosis [55,57].

**4.**  **Abdominal domain calculation and in utero monitoring**

Fetal MRI contributes to prenatal surgical planning by enabling estimation of abdominal domain and the volumetric relationship between the herniated organs and the fetal abdominal cavity, which may assist in anticipating feasibility of primary closure versus staged repair. In addition, MRI is particularly useful in late gestation when ultrasound is limited by fetal position, oligohydramnios, or acoustic shadowing, providing a stable anatomical reference for pre-delivery assessment and multidisciplinary planning.

MRI is typically performed in the late second to third trimester (approximately 24–36 weeks of gestation), when fetal size and anatomical resolution are optimal for detailed evaluation and perinatal management planning [55,56,57].

Although fetal MRI provides important complementary information in selected cases, accurate diagnosis and clinical decision making in complex fetal anomalies continue to rely on a comprehensive multimodal imaging approach, with ultrasound remaining the cornerstone of evaluation. 

Rare fetal conditions with major structural anomalies may present significant diagnostic and management challenges, even when they do not fall within the spectrum of classical anterior abdominal wall defects. In these settings, detailed prenatal ultrasonography, often complemented by advanced imaging techniques, is essential for accurate phenotypic characterization, differential diagnosis, and multidisciplinary perinatal counseling. The clinical value of meticulous imaging assessment and structured counseling in complex fetal malformations is further illustrated by reports of cephalo–thoraco–omphalopagus conjoined twins in triplet pregnancies, in which detailed ultrasound evaluation was pivotal for diagnosis, prognostic assessment, and perinatal management planning [58].

#### 5.3.2. Gastroschisis

Gastroschisis is typically diagnosed by ultrasound during the second-trimester anomaly scan, around 20 g.w.

1. Detailed anatomic survey

Gastroschisis is characterized by paraumbilical evisceration of bowel, typically right-sided, without a covering membrane and with freely floating intestinal loops in the amniotic cavity [59,60]. Targeted sonographic evaluation should assess bowel caliber, wall thickness, echogenicity, peristalsis, intra-abdominal bowel size, and the appearance of the extra-abdominal bowel loops. Real-time ultrasonography enables dynamic assessment of bowel motility and the appearance of the eviscerated intestinal loops throughout the examination, whereas color Doppler imaging may be used to evaluate mesenteric vascular perfusion when vascular compromise, volvulus, or evolving bowel ischemia is suspected. Serial ultrasound examinations combining real-time imaging and Doppler assessment may assist in monitoring disease progression and identifying fetuses at increased risk of complex gastroschisis who require closer surveillance and individualized delivery planning. Extra-abdominal bowel dilatation (>10–18 mm), progressive wall thickening, and non-free-floating loops have been associated with complex gastroschisis (atresia, stenosis, necrosis) and adverse neonatal outcomes [42,61,62]. Serial growth assessment is recommended due to the increased risk of fetal growth restriction and preterm delivery [63].

2. Assessment of cord insertion, liver herniation, and spinal alignment

The umbilical cord insertion is typically normal and separate from the defect, which is a key diagnostic feature distinguishing gastroschisis from omphalocele [59,60]. Liver herniation is absent; its presence should prompt reconsideration of the diagnosis. Spinal alignment is usually normal.

3. Fetal echocardiography

Routine fetal echocardiography is not mandatory in isolated gastroschisis due to the low incidence of associated structural cardiac defects; however, it is recommended when additional anomalies are suspected [61,64].

4. Genetic testing (CVS/amniocentesis)

Isolated gastroschisis has a low association with chromosomal abnormalities; therefore, invasive testing is not routinely indicated but should be offered if additional structural findings are identified [59,63].

#### 5.3.3. Omphalocele

1. Detailed anatomic survey

Omphalocele presents as a midline defect at the base of the umbilical cord with herniation of abdominal viscera into a membrane-covered sac [60,65]. Detailed assessment should document sac size, content (bowel only vs. liver-containing), sac integrity, and associated structural anomalies. Real-time ultrasonography facilitates dynamic assessment of bowel peristalsis within the omphalocele sac, while color Doppler imaging may assist in evaluating vascular perfusion of the herniated viscera, confirming the relationship between the sac contents and the umbilical vessels, and identifying associated vascular abnormalities when suspected. Extracorporeal liver herniation and large defect size correlate with increased morbidity and mortality [66]. Associated anomalies, particularly cardiac, central nervous system, skeletal, and genitourinary defects, occur in up to 50–70% of cases, necessitating comprehensive evaluation [64,66].

2. Assessment of cord insertion, liver herniation, and spinal alignment

The umbilical cord inserts at the apex of the sac, a defining feature [60,65]. Liver herniation is common and represents an important prognostic marker [66]. In cases with liver herniation, prenatal diagnosis of omphalocele with ultrasound can be made as early as 9–10 g.w., whereas in cases without liver herniation, reliable diagnosis is typically achieved after 12 weeks once physiological midgut herniation has resolved [60,65]. Evaluation of spinal alignment is required to exclude syndromic or complex body wall malformations.

3. Fetal echocardiography

Fetal echocardiography is strongly recommended in all cases due to the high prevalence of congenital heart disease, which significantly impacts survival and management [64,65].

4. Genetic testing (CVS/amniocentesis).

Omphalocele is frequently associated with other congenital anomalies, with cardiac defects reported in approximately 30–50% of cases and chromosomal abnormalities in about 20–30%, whereas central nervous system defects comprise a smaller proportion of associated anomalies [66]. Given the well-established association of omphalocele with aneuploidies and syndromic disorders, including trisomies 13, 18, and 21 and Beckwith–Wiedemann spectrum (BWS), invasive diagnostic testing represents the cornerstone of genetic evaluation and should be routinely offered following prenatal diagnosis [65,66].

Chorionic villus sampling (typically performed at 10–13 g.w.) [67] or amniocentesis (after approximately 15 g.w.) with chromosomal microarray analysis (CMA) is recommended for definitive cytogenetic diagnosis [68]. Compared with conventional karyotyping, CMA provides a higher diagnostic yield by detecting submicroscopic pathogenic copy number variants that may not be identified by standard cytogenetic analysis. In selected cases, particularly when CMA is normal but the prenatal phenotype is suggestive of abother syndromic condition, additional targeted molecular investigations, including testing for BWS, should be considered as part of the diagnostic work-up.

Non-invasive prenatal testing (NIPT) should be regarded exclusively as a screening tool for common aneuploidies and cannot replace invasive diagnostic testing in fetuses prenatally diagnosed with omphalocele. Although highly sensitive for trisomies 13, 18, and 21, NIPT cannot detect structural fetal anomalies, most pathogenic copy number variants, or many syndromic disorders, and false-positive or false-negative results may occur because of biological factors such as confined placental mosaicism or low fetal fraction. Consequently, a normal NIPT result does not exclude omphalocele or associated genetic conditions, and a positive result always requires confirmation by invasive diagnostic testing. Therefore, when an omphalocele is identified on prenatal ultrasound, pre-test genetic counseling followed by invasive diagnostic testing with CMA should be considered the standard diagnostic approach, with additional syndrome-specific testing performed when clinically indicated.

#### 5.3.4. Body Stalk Anomaly (BSA)

1. Detailed anatomic survey

BSA is typically diagnosed in the first trimester and is characterized by a large ventral body wall defect with evisceration of abdominal and often thoracic organs, severe kyphoscoliosis, and limb abnormalities [30,69]. The absence of a discrete abdominal wall and adherence of the fetus to the placenta may be observed, and three-dimensional ultrasound may aid in spatial characterization [69]. A recent retrospective study of 37 fetuses with limb–body wall complex further reinforces the feasibility of early prenatal diagnosis and highlights the value of systematic ultrasonographic assessment in improving phenotypic characterization, differential diagnosis, and parental counseling.

2. Assessment of cord insertion, liver herniation, and spinal alignment.

The umbilical cord is markedly shortened or absent, and the abdominal contents, including the liver, are usually exteriorized without a protective membrane [30]. Severe axial skeletal deformities, including kyphoscoliosis, are characteristic and essential for diagnosis [30,69].

3. Fetal echocardiography

Although BSA is generally considered lethal, echocardiographic assessment may be performed when technically feasible to document associated cardiac anomalies and assist in differential diagnosis [30,69].

4. Genetic testing (CVS/amniocentesis)

Chromosomal abnormalities are less consistently associated than in omphalocele; however, invasive testing may be considered on an individualized basis, particularly when the diagnosis is uncertain or when overlapping phenotypes are suspected [69].

**Figure 9:**
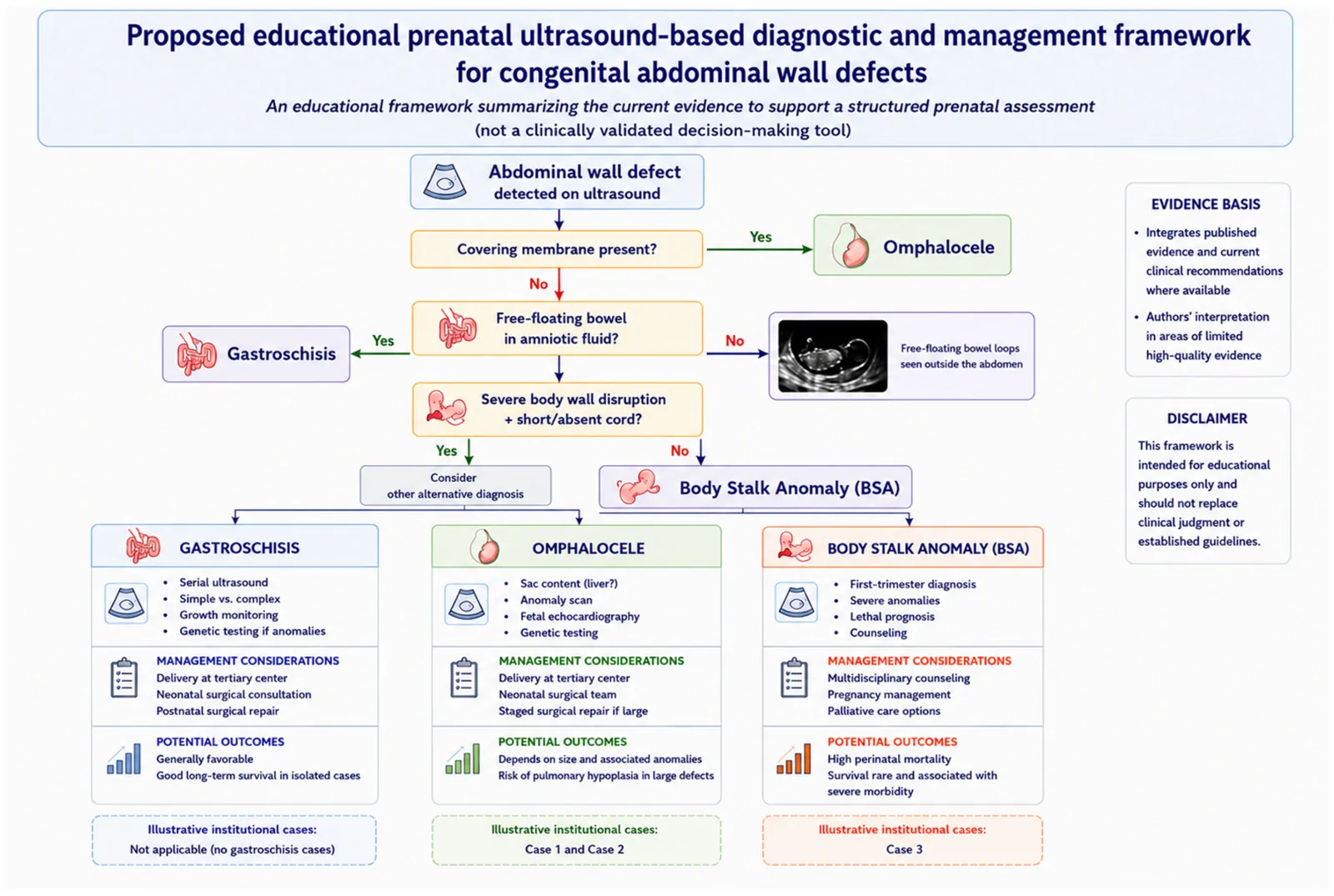
**Prenatal educational ultrasound-based diagnostic and management framework summarizing the current evidence for congenital abdominal wall defects.** The framework summarizes key prenatal ultrasound findings and management considerations synthesized from the reviewed literature, existing clinical recommendations where available, and the authors’ illustrative institutional cases. It is intended as an educational aid and narrative summary of the current evidence rather than a clinically validated decision-making tool.

To facilitate the practical application of the findings presented in this review and summarized in Table 4, we synthesized the evidence from the reviewed literature into a proposed prenatal ultrasound-based diagnostic and management framework (Figure 9). The illustrative institutional cases are presented as representative examples supporting the diagnostic pathways depicted in the framework. The framework integrates key diagnostic features and management considerations derived from published evidence and current clinical recommendations where available while reflecting the authors’ interpretation where high-quality evidence remains limited. It is intended as an educational summary of the current evidence rather than as a clinically validated decision-making tool.

### 5.4. Clinical Considerations

Non-invasive prenatal testing (NIPT) can complement the evaluation of CAWDs, but it cannot replace imaging. NIPT screens cell-free fetal DNA for common aneuploidies (trisomies 21, 18, and 13) and select sex chromosome abnormalities, with high sensitivity from 10 WGA. However, structural defects such as gastroschisis, omphalocele, or body stalk anomaly are not detectable by NIPT, as they arise from embryologic malformations rather than chromosomal aneuploidies. Thus, a normal NIPT result does not exclude CAWDs, and an abnormal result only indicates potential chromosomal risk, requiring confirmation by detailed ultrasound and, if indicated, invasive testing. NIPT is most informative when an omphalocele is identified, given its higher association with trisomies, yet ultrasound remains the diagnostic cornerstone, and positive NIPT findings should always be verified before clinical decisions are made.

### 5.5. Clinical Follow-Up During Pregnancy

The medical management of CAWDs is primarily determined by two major factors: the presence and severity of associated structural or chromosomal abnormalities and the size of the herniated mass. While termination of pregnancy (TOP) is strongly associated with complex genetic syndromes and severe structural anomalies, expectant management is focused on serial fetal surveillance aimed at optimizing gestational age at delivery and preventing progressive bowel or solid organ injury.

- Gastroschisis

Gastroschisis is typically an isolated paraumbilical defect with a normal fetal karyotype and a survival rate exceeding 90% in most series. Follow-up should include monitoring of fetal growth and bowel health using serial ultrasound, with particular attention to signs of bowel dilation or compromised perfusion that may predict intestinal complications. Ultrasound surveillance is often performed every 4 weeks prior to 28 weeks and every 2 weeks thereafter to detect adverse trends that could influence the timing of delivery. Additional assessment of amniotic fluid volume and fetal well-being is also recommended.

**Antenatal Decision Making**

- **Indications for TOP**

TOP is rarely indicated in gastroschisis and is generally exceptional (<0.1%), given the absence of consistent chromosomal associations. TOP may only be considered in extremely rare cases where gastroschisis is associated with severe additional lethal anomalies.

The size of the herniated bowel mass is not an indication for termination; however, disease severity is influenced by the extent of bowel exposure and the development of complex gastroschisis. Complex forms include bowel atresia, perforation, necrosis, or volvulus, which are associated with worse neonatal outcomes but are typically managed postnatally.

- **Expectant Management Protocol**

Expectant management is based on detailed serial ultrasound assessment of bowel morphology and amniotic fluid dynamics. Key parameters include bowel dilation, bowel wall thickening (reflecting inflammatory “peel” formation), and evidence of bowel matting or compromised perfusion.

In addition, surveillance should assess for progressive reduction in abdominal wall defect size (“closing gastroschisis”), which may lead to vascular compression of the mesenteric root and subsequent midgut ischemia, necrosis, or vanishing gastroschisis.

**Timing and Route of Delivery**

There is no universal indication for preterm delivery in gastroschisis. In uncomplicated cases, delivery is typically planned at 36–37 g.w. to reduce intestinal morbidity and optimize neonatal surgical outcomes. However, delivery timing should be individualized according to disease severity. Earlier delivery may be indicated in complex gastroschisis, fetal growth restriction, worsening bowel parameters (e.g., progressive bowel dilation, wall thickening or signs of ischemia), abnormal Doppler findings, or non-reassuring cardiotocography. Vaginal delivery is generally considered safe in uncomplicated (isolated/simple) gastroschisis and is not contraindicated in such cases, with cesarean section reserved for standard obstetrics indications. Prognosis in gastroschisis is generally favorable, with reported survival rates > 90% when delivered and managed in experienced centers [70].

- **Omphalocele**

Fetuses with prenatally diagnosed omphalocele require serial ultrasound assessments every 2–4 weeks to monitor fetal growth, evaluate cardiac function, quantify amniotic fluid (including polyhydramnios), and detect associated structural anomalies. Omphalocele is frequently associated with additional chromosomal aneuploidies, including trisomies 13, 18, and 21, as well as syndromic conditions such as Beckwith–Wiedemann syndrome and Pentalogy of Cantrell: retrospective cohort data report structural anomalies in >50% and chromosomal abnormalities in approximately 8% of cases, with prognosis largely determined by associated conditions [70]. Genetic testing (CVS or amniocentesis with microarray when indicated) and detailed fetal echocardiography should be offered to support prenatal counseling.

**Antenatal Decision Making**

- **Indications for TOP**

TOP is primarily determined by the presence of associated abnormalities rather than defect size alone. TOP is considered in cases of lethal aneuploidies (e.g., trisomy 13 or 18), severe congenital heart disease such as tricuspid atresia, or major central nervous system malformations. In non-isolated omphalocele cases, elective termination rates may reach up to 75%.

Defect size is a relevant prognostic modifier in omphalocele, particularly in cases of giant omphalocele (GO), commonly defined by herniation of more than 75% of the liver, which is associated with severe viscero-abdominal disproportion and increased risk of pulmonary hypoplasia, which significantly worsens outcome and may influence counseling regarding prognosis and management options.

- **Expectant Management Protocol**

In isolated omphalocele with normal karyotype and normal fetal echocardiography, expectant management is strongly recommended, with reported survival rates of 80–90%. Serial ultrasound surveillance is required to monitor fetal growth, assess integrity of the sac membrane, and detect intrauterine growth restriction.

**Timing and Route of Delivery**

Delivery should be individualized and is typically planned around 37–38 g.w. in uncomplicated cases, based on fetal growth and the presence of associated complications. However, timing should be tailored according to lesion characteristics and fetal condition. Earlier delivery may be considered in cases of giant omphalocele (GO), particularly when associated with liver herniation and severe viscero-abdominal disproportion, as well as in the presence of fetal growth restriction, abnormal Doppler or cardiotocography findings, progressive polyhydramnios, or other obstetric indications. Vaginal delivery is generally appropriated in cases of small, isolated omphalocele, whereas cesarean section may be considered in giant omphaloceles (GOs) to reduce the risk of sac rupture, liver injury, and dystocia. Preterm delivery is not routinely indicated in the absence of obstetric complications. Survival is highest in isolated cases and significantly lower in non-isolated omphalocele [70].

- **Body Stalk Anomaly (BSA)**

BSA is a rare, lethal defect characterized by a large abdominal wall defect with spine and limb anomalies and a very short or absent umbilical cord. Prenatal diagnosis should be confirmed by detailed morphologic imaging, typically in the first trimester, as early identification facilitates counseling. Chromosomal anomalies are not commonly associated, and the condition is incompatible with life, warranting sensitive counseling about prognosis and options, including TOP [30].

**Antenatal Decision Making**

Given the uniformly lethal prognosis of BSA, early confirmation of non-viability is essential. Counseling should be provided as soon as the diagnosis is established, as most cases are identified in the first trimester, allowing for timely discussion of pregnancy continuation versus termination of pregnancy according to parental choice and local legal and ethical frameworks.

Across all CAWDs, delivery should be planned at a center capable of multidisciplinary care, with obstetrics, neonatology, pediatric surgery, and genetics expertise. Cesarean section is not routinely required solely for omphalocele or gastroschisis, except in specific obstetric indications (e.g., giant omphalocele or severe complications), and mode/timing should be individualized based on fetal status and associated conditions [71]. Overall, the indication for TOP in CAWDs is primarily driven by the presence of co-existing genetic abnormalities or severe structural malformations, particularly in omphalocele, whereas expectant management and timing of delivery are guided by dynamic changes in the herniated mass, including its size and associated bowel or organ compromise.

In addition to antenatal decision making and delivery planning, standardized obstetric interventions aimed at reducing prematurity-related morbidity remain essential in the management of pregnancies complicated by CAWDs. Delivery planning should therefore be stratified according to lesion type and severity rather than fixed gestational age thresholds.

#### Antenatal Corticosteroids and Magnesium Sulfate Administration

In cases where preterm delivery is anticipated, standard obstetric protocols for fetal neuroprotection and respiratory maturation should be followed in pregnancies complicated by congenital abdominal wall defects. Antenatal corticosteroid administration (e.g., betamethasone) is recommended between 24 and 33 + 6 weeks of gestation to enhance fetal lung maturation and reduce the risk of respiratory distress syndrome (RDS), intraventricular hemorrhage, and necrotizing enterocolitis. Similarly, magnesium sulfate should be administered when early preterm delivery is expected, typically before 32–34 weeks of gestation, for fetal neuroprotection and reduction in the risk of cerebral palsy.

The presence of CAWDs, including gastroschisis and omphalocele, does not modify the indication for either intervention, as their benefits in improving prematurity-related outcomes remain applicable irrespective of the underlying abdominal wall defect. However, these therapies are particularly relevant in CAWD-associated complicated pregnancies due to the potential need for early delivery and complex neonatal surgical management.

### 5.6. Neonatal Care After Birth

Immediate postnatal management of CAWDs is crucial and should begin at delivery to prevent hypothermia, fluid loss, and infection. Newborns should be promptly stabilized in a thermoregulated environment, with exposed abdominal contents protected using sterile, warm, saline-soaked gauze and occlusive plastic wrapping or polyethylene bowel bags. Plastic bag wrapping has been shown to be an effective and simple intervention to reduce evaporative heat loss and prevent hypothermia, particularly in preterm or low-birth-weight neonates.

The effectiveness of occlusive plastic wrapping is particularly relevant in gastroschisis, which is characterized by the absence of a protective peritoneal covering over the exposed bowel loops. This lack of peritoneal protection results in direct exposure of the intestines to the external environment, leading to significant heat loss, fluid evaporation, and increased risk of infection. Consequently, immediate occlusive wrapping is essential to minimize these complications and stabilize the neonate prior to surgical intervention.

- **Gastroschisis**

In gastroschisis, primary surgical reduction with placement of the eviscerated bowel into the abdominal cavity is successful in approximately 50–80% of cases, depending on abdominal domain capacity and bowel edema, allowing closure without excessive intra-abdominal tension. When primary closure is not feasible, staged reduction techniques are required. A preformed silo is commonly used to facilitate gradual reduction of bowel contents while minimizing intra-abdominal pressure and reducing the risk of abdominal compartment syndrome. In selected cases, a Bogotá bag may also be used as an alternative silo technique to support staged reduction.

In cases of large abdominal wall defects, particularly giant omphalocele (GO), staged closure is often required due to limited abdominal domain and risk of cardiorespiratory compromise.

- **Omphalocele**

In omphalocele, surgical strategy depends on defect size: small to medium defects are generally managed with primary surgical closure, whereas large or giant omphaloceles typically require staged closure.

Early involvement of a multidisciplinary neonatal and pediatric surgical team is essential in all cases. Gastric decompression with a nasogastric tube should be performed to reduce bowel distension and minimize aspiration risk. Subsequent surgical planning should determine whether primary closure or staged reduction is appropriate, based on defect type, size, abdominal domain capacity, and overall neonatal stability. The main management drivers are summarized in Table 5. 

### 5.7. Parental Counseling and Ethical Considerations

Prenatal parental counseling is central to the management of fetal abdominal wall defects. Parents should be informed not only about the anatomical diagnosis but also about prognosis, potential postnatal interventions, long-term outcomes, and quality of life. Counseling should clearly differentiate between lethal anomalies, where survival is not possible, and correctable anomalies, where postnatal surgery can provide long-term survival with variable morbidity. Multidisciplinary teams should discuss surgical options, likely prognosis, and anticipated neonatal and developmental outcomes while also providing psychosocial support and guidance regarding continuation or termination of pregnancy [72].

Antenatal detection allows for early identification of associated structural and chromosomal anomalies, which are significantly more common in fetuses with omphalocele compared to gastroschisis. This information is critical for prenatal counseling and delivery planning [70].

### 5.8. Differential Diagnosis: Isolated vs. Complex vs. Lethal Defects

Accurate differential diagnosis is essential for parental counseling and perinatal management. Isolated defects, such as gastroschisis, involve only the abdominal wall and are generally surgically correctable with favorable prognosis. Complex defects, such as omphalocele with associated cardiac or other structural anomalies, carry a higher risk of morbidity and may require coordinated postnatal interventions. Lethal defects, such as BSA, are incompatible with life, characterized by severe abdominal wall defects, absent or rudimentary umbilical cord, and axial skeletal anomalies. Detailed morphological imaging, including assessment of the umbilical cord, limbs, spine, and associated anomalies, is critical to accurately classify defects and guide counseling, management, and delivery planning [30,71,72].

### 5.9. Body Stalk Anomaly as a Lethal Entity

Body stalk anomaly is a rare and uniformly lethal condition, often presenting in the first trimester. It is defined by a severe abdominal wall defect, absent or rudimentary umbilical cord, and associated spinal and limb abnormalities. Prenatal diagnosis should be confirmed through detailed morphologic imaging to provide accurate counseling and avoid unnecessary interventions. Parents should be informed of the universally poor prognosis, limited role of surgical or postnatal intervention, and options including pregnancy termination. Ethical considerations emphasize parental autonomy, psychosocial support, and planning for possible intrauterine demise or perinatal death. Multidisciplinary management ensures that families receive coordinated care and guidance throughout the pregnancy [22,30].

## 6. Conclusions

Early and accurate differentiation between isolated, complex, and lethal abdominal wall defects enables tailored prenatal surveillance, optimal delivery planning, and informed parental counseling. While non-invasive prenatal testing can identify associated chromosomal risks, detailed ultrasound remains essential for diagnosis and perinatal management. Multidisciplinary follow-up ensures timely interventions for correctable defects and provides comprehensive support for families facing lethal anomalies such as body stalk anomaly, ultimately improving outcomes and guiding ethically sound decision making.

## Figures and Tables

**Table 1:** **Summary of case series of congenital abdominal wall defects**.

Case	Diagnosis	GA at Diagnosis	Key Ultrasound Findings	Risk Classification	Management	Outcome
1	Omphalocele + trisomy 18	20 weeks	Membranous sac, associated anomalies	Non-isolated (high risk)	Genetic testing, termination	Pregnancy terminated
2	Omphalocele + IUFD	2nd trimester	Omphalocele, hydrothorax, Spalding sign	Complex	Expectant management	IUFD
3	Body stalk anomaly	1st trimester	Large defect, absent cord, severe anomalies	Lethal	Counseling, termination	Pregnancy terminated

**Table 2:** **Summary of epidemiological characteristics of major abdominal wall defects. Prevalence estimates may vary depending on inclusion of terminations of pregnancy and early prenatal detection**.

Condition	Prevalence (per 10,000 Births)	Approx. Incidence	Sex Distribution	Key Epidemiological Associations	Prognosis
Gastroschisis	1.5–4.5 [5,6,7,8]	~1 in 2000–4000 [5,7]	M = F [11]	Young maternal age (<20), higher in Hispanic populations, singleton pregnancies [11]	Generally favorable (isolated cases) [11]
Omphalocele	1.5–3.6 [7,8]	~1 in 3000–5000 [7]	Slight M > F (variable) [7,11]	Strong association with chromosomal abnormalities (trisomies 13, 18, and 21) and syndromes [23]	Depends on associated anomalies [7,23]
BSA/LBWC	<1 [12,22]	~1 in 10,000–40,000 [12,22]	No clear pattern [12,22]	Sporadic, severe developmental disruption [12,22]	Lethal (in most cases) [12,22]

**Table 3:** **Classification of omphalocele**.

Classification Type	Subtype	Definition	Clinical Significance
**By size/content**	Small omphalocele	Defect containing bowel only	Better prognosis
	Giant omphalocele	Defect containing liver ± other organs (≥5 cm in diameter)	Risk of pulmonary hypoplasia, complex repair
**By associated anomalies**	Isolated	No associated anomalies	Favorable outcome
	Non-Isolated	Structural/chromosomal anomalies present	Major determinant of mortality (c.f.t.r. Case 1)
**By sac integrity**	Intact sac	Membrane preserved	Protection of viscera
	Ruptured sac	Membrane disrupted	Increased risk of infections and complications

“c.f.t.r.”, meaning “case file to refer to”.

**Table 4:** **Abdominal wall defects: overview**.

Feature	Gastroschisis	Omphalocele	Body Stalk Anomaly (BSA)
**Typical location**	Right-sided paraumbilical defect	Midline defect at umbilical ring	Large thoracoabdominal defect with absent or extremely short umbilical cord
**Embryologic basis**	Likely related to involution of the right umbilical vein and failure of ventral wall	Failure of midgut return and/or incomplete lateral body wall folding	Early failure of embryonic folding and ventral body wall formation
**Covering membrane**	None; bowel exposed to amniotic fluid	Present; viscera enclosed in a sac	None; viscera adherent to placenta or exposed
**Associated anomalies**	Usually isolated; bowel complications (atresia, perforation) possible; chromosomal anomalies rare	High rate of cardiac defects, chromosomal abnormalities, and syndromic associations	Severe multisystem anomalies (spine, limbs, urogenital, and gastrointestinal); uniformly lethal
**Characteristic variants**	Complex gastroschisis; closing/closed gastroschisis; vanishing gastroschisis	Giant omphalocele; hernia into the cord	Limb–body wall complex (LBWC)
**Prenatal ultrasound findings**	Free-floating bowel; separate cord insertion; bowel thickening/edema; liver herniation uncommon	Membranous sac; cord inserts into sac; frequent liver herniation	Large defect with eviscerated organs, severe scoliosis, and short/absent cord
**Mode of delivery**	Vaginal or cesarean depending on obstetric factors	Vaginal for small defects; cesarean often preferred for large defects or liver herniation	Delivery route based on maternal indications; prognosis uniformly poor
**Timing of delivery**	Late preterm to early term (≈35–38 weeks)	Term when feasible	No standardized timing due to lethality
**Immediate postnatal care**	Protect exposed bowel; maintain temperature; avoid umbilical venous access; gastric decompression	Protect sac; similar stabilization measures as gastroschisis	Palliative care due to non-survivable prognosis

**Table 5:** **Summary of management drivers**.

CAWD Type	Mass Size Impact on Management	Associated Anomalies Impact on Management	Primary Delivery Consideration
**Gastroschisis**	Large mass or dilation monitors for volvulus/atresia; shrinking mass risks ischemic closure.	Rarely associated with genetic defects; termination is exceptional (<1%).	Prevent progressive chemical/ischemic bowel damage.
**Omphalocele**	Giant size (>75% liver) dictates cesarean delivery; small defects can safely go vaginal.	High aneuploidy/syndrome link; dictates high termination rate (up to 75%) if complex.	Avoid sac rupture and hepatic trauma.

## Data Availability

Data presented in this study are available on request from the corresponding author due to privacy concerns.

## Abbreviations

The following abbreviations are used in this manuscript:

CAWDs	Congenital Abdominal Wall Defects
BSA	Body Stalk Anomaly
LBWC	Limb–Body Wall Complex
g.w.	Gestational Weeks
IUFD	Intrauterine Fetal Demise
CVS	Chorionic Villous Sampling
NIPT	Non-invasive Prenatal Testing
MRI	Magnetic Resonance Imaging
TOP	Termination of Pregnancy
